# Towards Sustainable Direct Recycling: Unraveling Structural Degradation Induced by Thermal Pretreatment of Lithium‐Ion Battery Electrodes

**DOI:** 10.1002/cssc.202400727

**Published:** 2024-09-13

**Authors:** Shuaiwei Liu, Oleksandr Dolotko, Thomas Bergfeldt, Michael Knapp, Helmut Ehrenberg

**Affiliations:** ^1^ Institute for Applied Materials-Energy Storage Systems (IAM-ESS) Karlsruhe Institute of Technology (KIT) Hermann-von-Helmholtz-Platz 1 D-76344 Eggenstein-Leopoldshafen, Karlsruhe Germany; ^2^ Helmholtz-Institute Ulm for Electrochemical Energy Storage (HIU) P.O. Box 3640 D-76021 Karlsruhe Germany; ^3^ Institute for Applied Materials Applied Materials Physics (IAM-AWP) Karlsruhe Institute of Technology (KIT) Hermann-von-Helmholtz-Platz 1 D-76344 Eggenstein-Leopoldshafen, Karlsruhe Germany

**Keywords:** Direct recycling, Spent lithium-ion battery, Thermal pretreatment, Structural degradation

## Abstract

As the demand for lithium‐ion batteries (LIBs) continues to increase, there is a growing focus on recycling these battery wastes. Among the existing recycling methods, direct recycling is considered a promising approach, because it allows waste to be returned directly to production. One crucial step in this process is the pretreatment, which involves separating the active materials from the current collector. Thermal treatment provides a feasible and effective approach for achieving this separation. Nonetheless, concerns persist regarding the potential impacts of this process on the structure. This study aims to examine the effects of thermal treatment on the separation efficiency and crystal structure of fresh and cycled NMC (LiNi_0.6_Co_0.2_Mn_0.2_O_2_) cathodes and graphitic anodes, under various atmospheres and temperatures. The results reveal that an air/oxygen atmosphere facilitates complete separation of cathode materials from aluminum with minimal structural degradation and at lower temperatures compared to other atmospheres. For graphite, thermal treatment under argon, nitrogen and hydrogen demonstrates good structural stability. However, for cycled anodes, the desired separation is not achieved due to the possible interface adhesion that occurs during cycling and heating. Additionally, compared to fresh materials, cycled materials experience more pronounced structural degradation during thermal treatment.

## Graphical abstract

Direct recycling is emerging as a promising option for the next generation of recycling technology. Thermal pre‐treatment offers a feasible way for this technology, enabling the effective separation of active materials from the current collector. This work examines the effects of thermal treatment on the separation efficiency and crystal structure of NMC622 cathode and graphite anode.

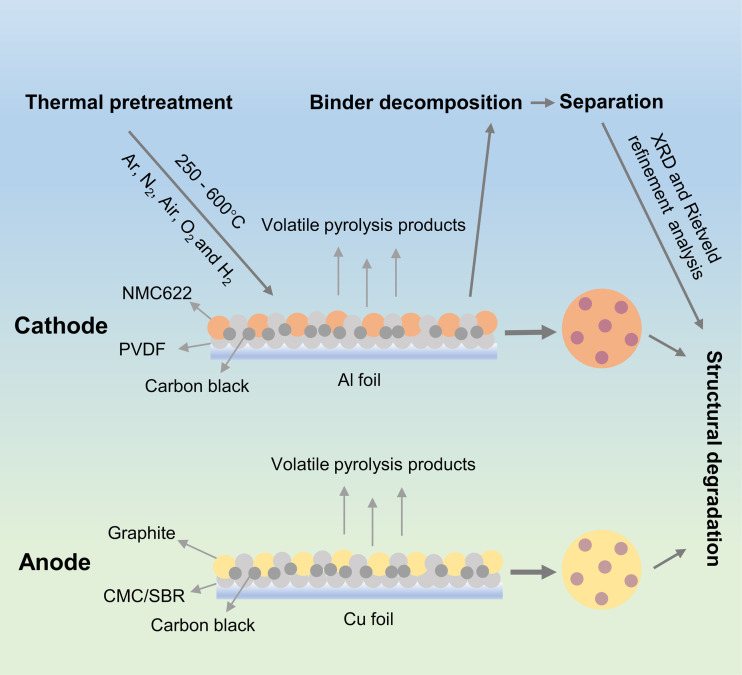

## 1 Introduction

The lithium‐ion battery (LIB) has experienced a tremendous growth since Sony released the first commercial LIB in 1991.^[1]^ The continually rising demand for LIBs in the electric vehicles (EVs) and consumer electronics brings significant concerns to the resources and the environmental sustainability of spent LIBs.^[2]^ Transition metals (Ni, Co, and Mn) and the flammable organic electrolytes contained in spent LIBs are highly harmful to the environment and human health under improper disposal. Furthermore, the rising cost of raw materials aggravates the concerns regarding the supply chain for LIBs.^[4]^ In this context, recycling of spent LIBs emerges as a potentially viable solution to mitigate such risks and promote resource sustainability.

Currently, the industrial recycling technologies employed for LIBs primarily consist of hydrometallurgy and pyrometallurgy methods.^[5]^ These technologies mainly focus on recovering the battery cathode′s most valuable metallic components (e. g., Li, Co and Ni). The development of EV batteries has resulted in a gradual shift towards high‐nickel and ultra‐nickel NMC materials (LiNi_*x*_Co_*y*_Mn_*z*_O_2_, *x*+*y*+*z*=1) for cathodes.^[7]^ Such transition poses a challenge for conventional recycling technologies, as the reduced economic return may no longer be sufficient to offset the high processing costs involved in recycling end‐of‐life LIBs.^[8]^ Additionally, the extraction and recovery of the valuable components in these processes are based on destroying the cathode materials, generally needing high amounts of energy and chemicals, and possibly causing environmental concerns.^[9]^ Direct recycling is a non‐destructive strategy for the spent cathodes and anodes scrape of LIBs, which has generated increasing interest in recent years.^[10]^ The focus is on restoring the damaged structure of the materials and compensating for the loss of elements, which allows the electrochemical properties of the regenerated materials to be restored to their original state.^[12]^ Nevertheless, one of the key challenges for this technology is to effectively separate the active materials in a non‐destructive manner while ensuring efficient control of impurities.^[13]^

The present separation technologies can be mainly divided into mechanical separation, dissolution, and thermal treatment.^[14]^ (1) Mechanical separation encompasses various processes, including crushing, sieving, magnetic separation, flotation, and others.^[15]^ Current industries widely employ this technology because it is capable of large‐scale application. However, controlling the impurity levels within an acceptable range in the obtained active materials poses a challenge, making it difficult to recover a satisfactory electrochemical performance of the recycled materials. (2) Dissolution refers to the process of using solvents to dissolve the binder, resulting in the separation of active materials. The most common solvents are N‐methyl pyrrolidone (NMP),^[18]^ dimethyl formamide (DMF),^[19]^ and dimethylacetamide (DMAC),^[20]^ which can effectively dissolve the polyvinylidene fluoride (PVDF) and separate the active materials from aluminum (Al) or copper (Cu) foil. This strategy effectively helps in preventing the inclusion of impurities. However, it is important to note that the deteriorated interface between active materials and current collector,[^13^, ^21^] as well as the formation of the cathode/anode electrolyte layer (CEI/SEI)^[22]^ after prolonged cycling, will hinder the dissolution of the binder, leading to a slow dissolving kinetics. Moreover, the high cost and toxicity associated with most organic solvents pose challenges for the large‐scale application of this technology. (3) Thermal treatment decomposes the binder at high temperatures, realizing the separation of active materials.^[23]^ It has the merits of simplicity, high efficiency, and the capability to control impurities. From the perspective of direct recycling, this technology is considered a promising approach for obtaining active materials. Previous works reflect that thermal treatment could further induce the structure degradation of active materials, which could bring difficulties in the subsequent re‐lithiation and structure repairment process,[^14^, ^25^] yet an investigation that systematically reveals structural changes after thermal treatment is still absent.

Herein, thermal treatment is employed to separate the active materials from aluminum and copper current collectors, where the connection between separation efficiency and temperature under different atmospheres is established. XRD analysis is utilized to examine structural changes of materials after thermal treatment. In addition, the visualization of structural information obtained using Rietveld refinement allowed us to find the suitable conditions that minimize structure degradation. By comparing fresh and cycled electrode materials, this study examines the effect of long‐term cycling on the separation process and the degradation of the structure. This work gives some new insights into the mechanism of structure evolution of electrode materials upon the thermolysis and provides guidance for the pretreatment process before direct recycling, devoting to the further development of future battery recycling.

## Methods

### Materials

Battery electrodes were provided by the KIT Battery Technology Center (KIT‐BATEC).^[27]^ The cathodes in this study were composed of NMC622 (LiNi_0.6_Co_0.2_Mn_0.2_O_2_), PVDF binder, and conductive carbon additive in a weight ratio of 96.0 : 2.0 : 2.0. The anodes for this study were composed of graphite, binder consisting of sodium carboxymethylcellulose and styrene‐butadiene rubber latex (CMC/SBR) and conductive additive carbon black in a weight ratio of 96.0 : 2.8 : 1.2. The current collectors of cathode and anode are aluminum and copper foil respectively. Fresh cathodes and anodes along with ceramic coated polyethylene terephthalate fabric (CC‐PET) as a separator, were assembled using 1 M LiPF_6_ in EC, DMC and VC as additives in the electrolyte to build multi‐layer pouch cells, resulting in a nominal capacity of 12 Ah.

The cells underwent 2100 cycles at a 1 C charge and discharge rate within the voltage range of 3.0–4.2 V until their capacity reached approximately 81 % of their initial level, indicating the end of their useful life. Subsequently, the LIBs were deeply discharged to 0 V, short‐circuited, and disassembled to extract the cycled cathodes and anodes for further analysis.

### Delamination Process

The fresh and cycled electrodes were cut into round pieces with a radius of 18 mm. These samples were then placed in the center of a gas flow furnace to undergo thermal treatment at various temperatures and atmospheres. A 55 l min^−1^ gas flow rate was maintained for experiments in argon and nitrogen atmospheres. The heating and cooling rates were set at 5 °C/min, and the samples were held at the highest temperature for 2 hours. After the thermal treatment process, the fresh and cycled electrodes were immersed in 15 ml of solvent and subjected to sonication for 10 seconds. After sonication, the obtained active materials, as well as the aluminum and copper foils, were dried overnight in an oven set at a temperature of 80 °C. This ensured the complete removal of any remaining solvent and moisture from the samples, preparing them for further analysis and characterization.

### Analytical Method

The weight differences of electrodes before (*M*_1_) and after (*M*_2_) the peeling‐off process, which involved the complete removal of the active material, were measured and denoted as *M_0_* (M_0_=*M*_1 –_ *M*_2_), representing 100 % delamination efficiency. Then, the electrodes before (*M_x1_*) and after (*M_x2_*) subjecting the electrodes to thermal treatment, sonication, and drying processes, were measured, and the difference in weight was denoted as *M_x_* (M_*x*_=*M*_*x*1 –_ *M*_*x*2_). Using these measurements, the separation efficiency (η) was determined by employing the following equation:

$$\eta \;\left(\%\right)=\frac{M_{x}}{M_{0}}\times 100\;\%$$

### Materials Characterization

Crystal structure characterization was carried out using the X‐ray powder diffraction (XRD) on a STOE Stadi P powder diffractometer with monochromatic Cu−Kα_1_ radiation (λ=1.54056 Å) in transmission geometry. The XRD measurements were performed at room temperature with a 0.015° 2θ step between 10 and 70 degrees of 2θ. The Kapton film′s presence visibly adds amorphous‐like background in the XRD patterns at 10°<2θ<17°.

The structural data were acquired through Rietveld refinement performed in the FullProf software package.^[28]^ The background of all the diffraction patterns was fitted using linear interpolation between selected data points in regions with no reflections present. The Thompson‐Cox‐Hastings pseudo‐Voigt function was used for the reflection profile shape description. The scale factor, lattice parameter, fractional coordinates of atoms, their overall isotropic displacement (temperature) parameter, zero angular shift, profile shape parameters, and half‐width (Caglioti) parameters were allowed to vary during fitting.

The microstructural properties of the materials were studied by using scanning electron microscopy (SEM). Images of the fresh and cycled materials were collected using the MERLIN Scanning Electron Microscope from Carl Zeiss. The elements except oxygen of the materials were analyzed by Inductively Coupled Plasma Optical Emission Spectrometer (ICP‐OES) and oxygen was analyzed by Thermal Extraction with Carrier Gas (TECG**)**.

## 2 Results and Discussion

### 2.1 Separation Efficiency Investigation

Photographs of the fresh and cycled cathodes used in the current study are shown in Figure S1a‐b. The surface of the fresh cathode is smooth and clean, while the surface of cycled cathode appears rougher. Additionally, the presence of white dots on the cycled cathode suggests the possible loss of active material, revealing the underlying bare aluminum foil. The XRD results indicate that the fresh and cycled materials can be indexed to the hexagonal *α*‐NaFeO_2_ layered structure (*R‐3 m* space group) (Figure S1c–f). The noticeable separation of 006/102 and 108/110 reflections represents a well‐ordered layered structure in both cases.^[29]^ There is no obvious difference between these two XRD results, suggesting the bulk phase is probably not affected much after cycling. The ratio of I_003_/I_104_ (peak intensity) for fresh and cycled cathodes are 1.60 and 1.68, respectively. The cycled material shows a slightly higher ratio of I_003_/I_104_, which is consistent with previously published studies.^[30]^ This phenomenon could be attributed to the preferred orientation of certain facets after microstructure changes upon cycling.^[32]^ Moreover, the SEM results indicate that the particle breakage in the cycled cathode is significantly more severe compared to the fresh cathode (Figure S1g–n). This microstructure change can be attributed to the repeated expansion and contraction of the cell volume upon cycling, as well as the corrosive effects of the electrolyte on the surface and interface of materials.^[33]^ Figure S2a‐b displays the appearance of the fresh and cycled anode. The stains on the surface of the cycled anode could be related to the corrosion of the electrode after its opening and exposure to air or surface changes upon battery cycling. The XRD results suggest that the fresh and cycled anode materials retain a hexagonal layer structure (*P63/mmc* space group) of graphite (Figure S2d‐e). The two samples exhibit similar morphology (Figure S2f–k).

**Figure 1:**
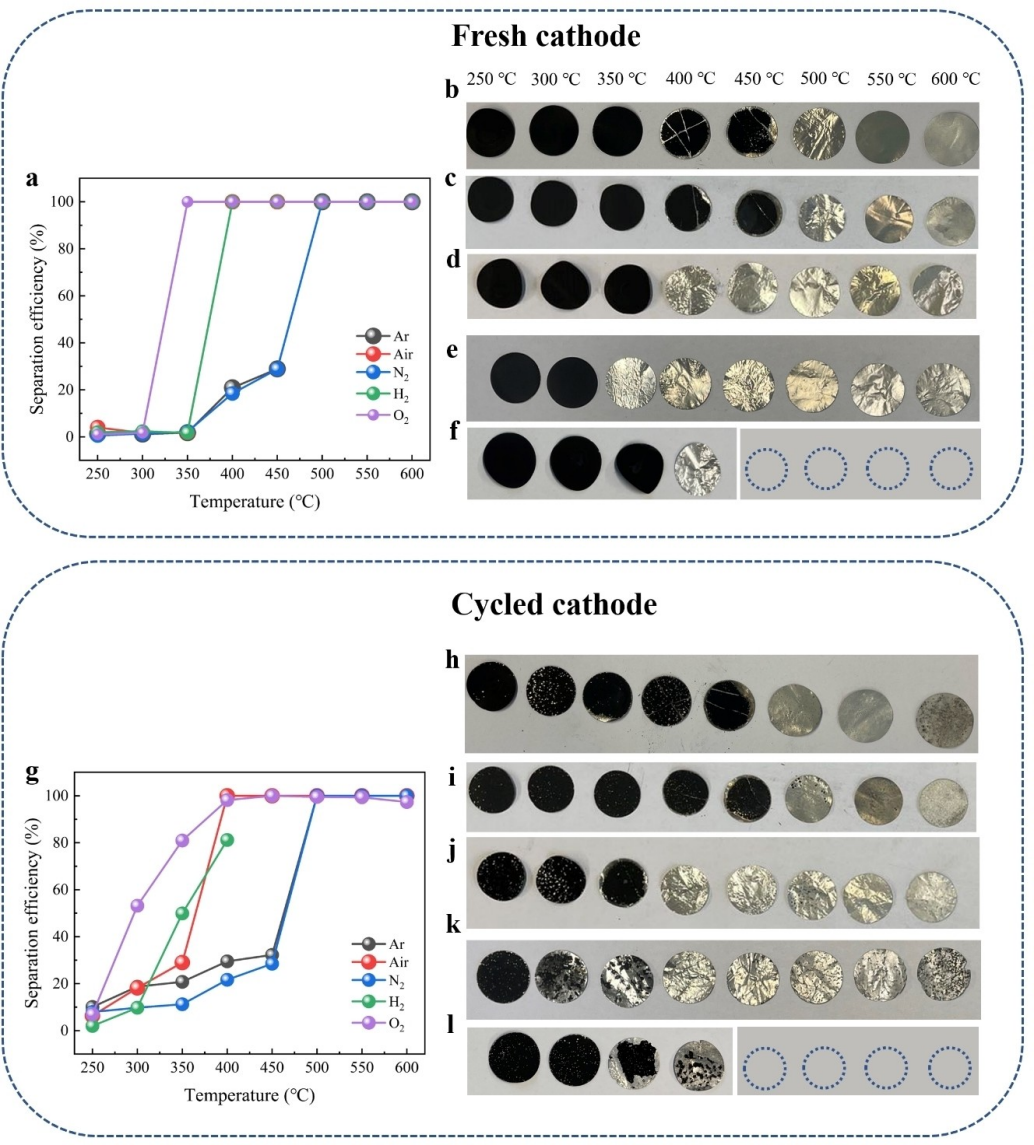
(a) Separation efficiency of active materials after thermal treatment under different atmospheres and temperatures for fresh cathodes (the separation efficiency under air/red achieves 100 % after 400 °C, but it is overlaid by other colors); Electrode state of fresh cathodes after thermal treatment with different temperatures under (b) argon, (c) nitrogen, (d) air, (e) oxygen and (f) hydrogen (empty circles represent that the electrodes have been transformed into powder after sonication). (g) Separation efficiency of active materials after thermal treatment under different atmospheres and temperatures for cycled cathodes; Electrode state of cycled cathodes after thermal treatment with different temperatures under (h) argon, (i) nitrogen, (j) air, (k) oxygen and (l) hydrogen.

The separation efficiency of cathode material from aluminum foil under different temperatures and atmospheres is shown in Figure 1a‐l. For the fresh cathodes, separation begins at 400 °C under argon, reaching 100 % at 500 °C (Figure 1a‐b). Under nitrogen, the results are similar, with 100 % efficiency at 500 °C, indicating that nitrogen, like argon, simply provides an inert environment (Figure 1c). The separation of the cathode is primarily due to the decomposition of PVDF. Prior studies have shown that PVDF begins to decompose at around 400 °C in an inert atmosphere, undergoing a gradual decomposition process.^[34]^ This decomposition releases gas phases and leaves about 20–25 wt % of carbon from PVDF in the final product. The volatile pyrolysis products mainly include HF gas and fluorocarbon chain fragments, resulting from the cleavage of the polymer chain and hydrogen transfer. As the temperature increases to 400 °C under inert conditions (such as argon and nitrogen), the separation efficiency improves, reaching 100 % at 500 °C. This temperature range corresponds to the onset, continuous progression, and complete decomposition of PVDF. Furthermore, studies have shown that an oxygen atmosphere can promote the decomposition of PVDF by forming oxygenated carbon compounds, which enhance reactivity.^[37]^ In our study, separation efficiency reaches 100 % at 400 °C in air and 350 °C in oxygen, both lower than the temperatures required under argon and nitrogen (Figure 1d‐e). This indicates that the presence of oxygen facilitates PVDF decomposition, allowing the pyrolysis process to occur at lower temperatures. With adequate holding time, the onset and complete decomposition of PVDF under both air and oxygen can occur at the same temperatures. Overall, the separation of cathode material from aluminum in these atmospheres is primarily driven by the decomposition of PVDF.

An interesting phenomenon is observed in a hydrogen atmosphere. Although hydrogen does not promote the decomposition of PVDF and may even inhibit it to some extent, 100 % separation efficiency is achieved at 400 °C (Figure 1f). This suggests that factors beyond the decomposition of PVDF influence the separation process in hydrogen. We attribute this additional factor to significant volume changes within the cell due to structural degradation. This structural degradation weakens the bonds among active material, PVDF, and aluminum, thus promoting separation. Additionally, temperatures above 400 °C lead to the brittleness of aluminum. This brittleness arises from the phase transformation of transition‐metal oxide into transition‐metal alloy, causing the aluminum foil to turn into powder during the sonication process. The more detailed explanation for these findings will be provided in the *Structural Stability Investigations* section.

For cycled cathodes, the separation efficiency reaches 100 % at 500 °C under argon and nitrogen (Figure 1g–i)., and 100 % at 400 °C under air (Figure 1j), similar to the fresh electrode. However, in an oxygen atmosphere, the separation efficiency at 350 °C is approximately 80 %, which differs from the fresh cathode (Figure 1k). This discrepancy can be attributed to the corrosion of current collector and the deteriorated interface between the active material and aluminum due to cycling.^[21]^ As observed, the aluminum in the cycled cathode shows more pronounced corrosion compared to the fresh cathode once active materials are removed. This corrosion leads to challenges in separating some particles effectively. Notably, at 300 °C under oxygen, cycled cathode demonstrates significantly higher separation efficiency than the fresh cathode. Similar trends are observed at both 300 and 350 °C under air, where the separation efficiencies of the cycled cathode surpass those of the fresh cathode. These increases in separation efficiency can likely be linked to the localized degradation of PVDF, caused by long‐term cycling. Previous studies have shown that the degradation of PVDF during cycling involves processes such as dehydrofluorination and the formation of carbon‐carbon double bonds.^[38]^ These reactions can be described as:

$$\left(1\right):\;{Li\;}^{+}-\left[CH_{2}-CF_{2}\right]-\;\to -\left[CH=CF\right]-\;+LiF+\frac{1}{2}H_{2}$$

$$\left(2\right):\;-\left[CH_{2}-CF_{2}\right]-\;\to -\left[CH=CF\right]-\;+HF$$

Research on the thermal stability of PVDF indicates that the stability is primarily provided by the C−F bonds, while its thermal degradation is driven by the formation of carbon‐carbon double bonds.^[40]^ Consequently, the degradation of PVDF upon cycling could render its carbon content more prone to oxidation, enhancing separation efficiency at lower temperatures under air and oxygen. In contrast, under inert atmospheres like argon and nitrogen, the decomposition of PVDF predominantly occurs through a gradual self‐lysis process. Therefore, the temperature required for complete separation remains consistent, and there is no significant increase in separation efficiency at lower temperatures compared to air and oxygen.

Under a hydrogen atmosphere, the separation efficiency does not reach 100 % within the temperature range, displaying a slightly lower efficiency at 400 °C when compared to fresh cathodes (Figure 1l). This can contribute from the deteriorated interface between the active material and aluminum as well. Moreover, it is observed that within the temperature range of 250 to 350 °C under different atmospheres, the separation of cycled cathodes (separation efficiency between 8 % and 20 %), although low, exceeds that of fresh cathodes (separation efficiency close to zero). As we know, CEI layer is formed on the surface of cathode particles during cycling, composed of various chemical compounds due to the reaction between the electrolyte and the cathode material.^[42]^ The weight between 250 and 350 °C can be predominantly attributed to the decomposition of these chemical compounds that remain on the electrode surface.

**Figure 2:**
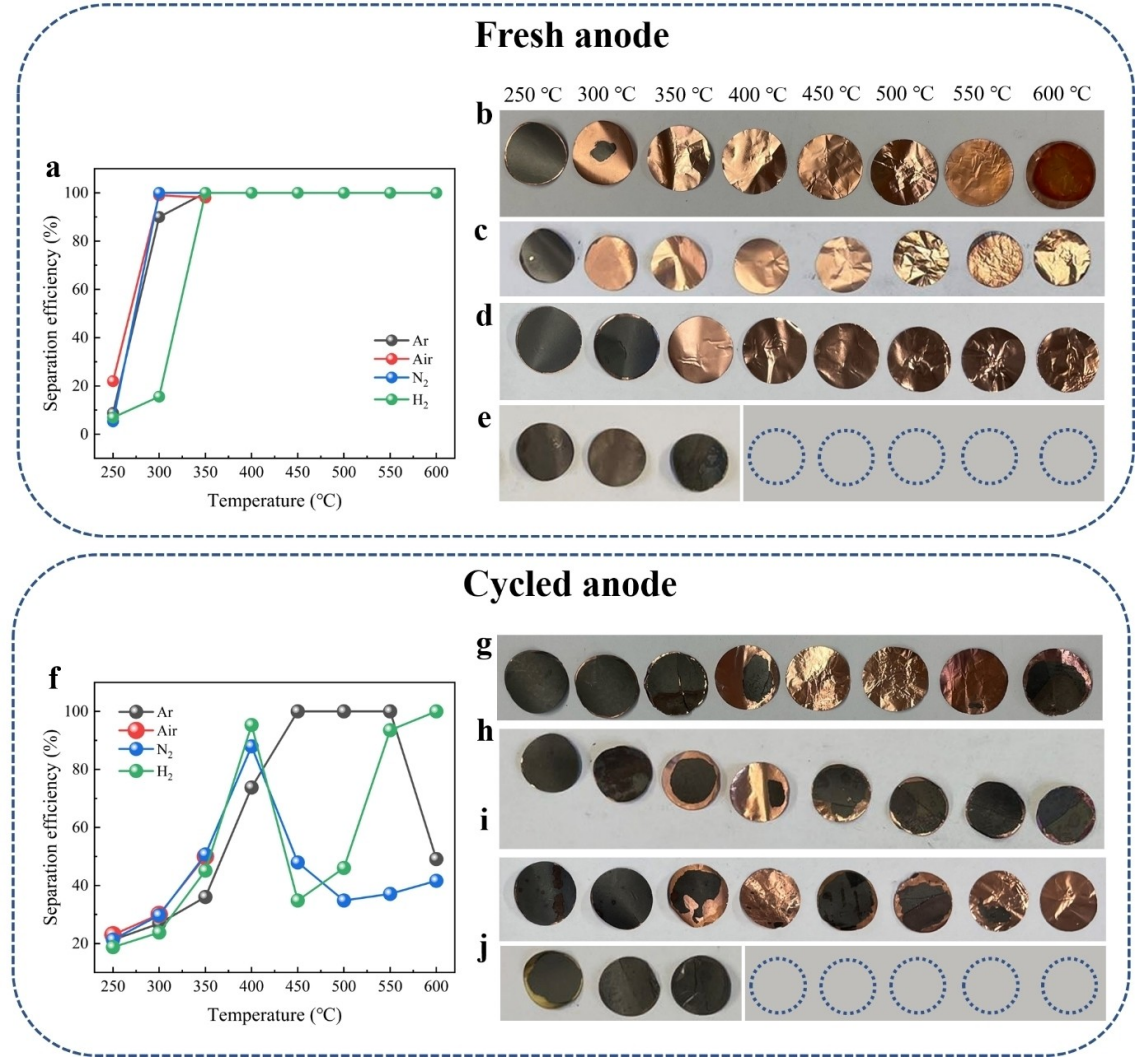
(a) Separation efficiency of active materials after thermal treatment under different atmospheres and temperatures for fresh anodes (the separation efficiencies under Ar/N_2_ from 350 to 600 °C are 100 %, but it is overlaid by other colors); Electrode state of fresh anodes after thermal treatment with different temperatures under (b) argon, (c) nitrogen, (d) hydrogen and (e) air (empty circles represent that the electrodes transform into powder after sonication). (f) Separation efficiency of active materials after thermal treatment under different atmospheres and temperatures for cycled anodes; Electrode state of cycled anodes after thermal treatment with different temperatures under (g) argon, (h) nitrogen, (i) hydrogen and (j) air.

Figure 2a–j displays the separation efficiency of anode materials from copper foil under different temperatures and atmospheres. Unlike the cathode material, graphite does not undergo drastic lattice changes or phase transformation during the thermal treatment, as will be elucidated later. In this case, the separation of graphite from copper foil is mainly realized by the decomposition of the binder, composed of CMC and SBR. Prior works have demonstrated that the initial decomposition temperatures of CMC and SBR are both approximately 300 °C,^[43]^ respectively. As a result, under argon and nitrogen atmospheres with a holding time of 2 h, the detachment of active materials from the copper foil begins at 300 °C for fresh anode (Figure 2a–c), where the separation efficiency can be already close to100 %. However, when exposed to a hydrogen atmosphere, the separation efficiency at 300 °C is around 15 % and the full separation takes place at 350 °C (Figure 2d), suggesting that the reducing atmosphere inhibits the decomposition of CMC/SBR to some extent. When heated in air, although the separation efficiencies of the fresh anode are close to 100 % at 300 and 350 °C, the copper foils undergo substantial oxidation, which becomes more pronounced with increasing temperature (Figure 2e). This oxidation eventually leads to copper embrittlement, causing it to break into powder during the sonication process. Similarly, the cycled anode exhibits significant oxidation and embrittlement of the copper foil under air, but with lower separation efficiency at 300 °C and 350 °C compared to fresh anodes (Figure 2j). In addition, our findings reveal that under argon, nitrogen and hydrogen atmospheres, in some cases, the separation efficiency of cycled anodes at higher temperatures would be lower than that of fresh anodes (Figure 2f‐i). For instance, despite the displayed separation efficiency being close to 100 % under argon from 450 to 550 °C, further experiments (Figure S3a–f) suggest that structural changes, material degradation, and binder issues can lead to reduced separation efficiency in cycled anodes. In other words, due to the varied states of cycled anodes, different separation efficiencies were observed at the same temperatures. Similar to cathodes, anode particles form a SEI layer on their surface during cycling as well.^[45]^ Additionally, copper corrosion and interface degradation would also occur upon cycling. Therefore, we would ascribe the difference in separation efficiency at the same temperatures to the decomposition of SEI and the CMC/SBR binder upon thermal treatment. Such decomposition induces chemical alterations at the interface contact, reinforcing the adhesion between the graphite and copper foil.

### 2.2 Structural Stability Investigations of Cathodes

#### 2.2.1 Cathodes under Argon

**Figure 3:**
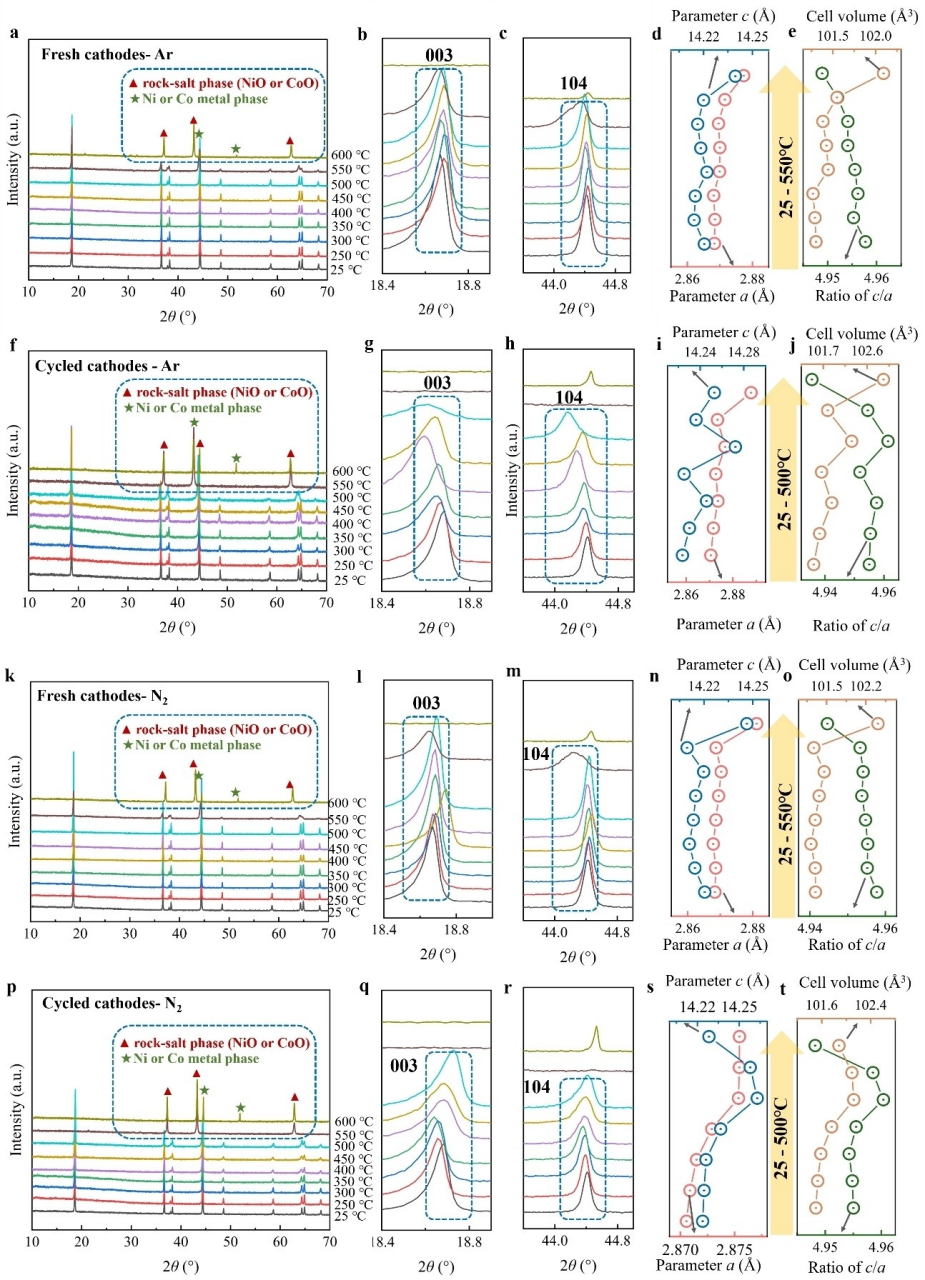
***Cathodes under argon***. (a) XRD pattern of fresh cathodes under argon from 25 to 600 °C, and its area of (b)18.4–19.0°, (c) 43.8–45.0°; The corresponding (d) parameters *a* and *c*, (e) ratio of *c/a* and cell volume from 25 to 550 °C. (f) XRD pattern of cycled cathodes under argon from 25 to 600 °C, and its enlarged area of (g)18.4–19.0°, (h) 43.8–45.0°; The corresponding (i) parameters *a* and *c*, (j) ratio of *c/a* and cell volume from 25 to 500 °C. ***Cathodes under nitrogen***. (k) XRD pattern of fresh cathodes under nitrogen from 25 to 600 °C, and its enlarged area of (l)18.4–19.0°, (m) 43.8–45.0°; The corresponding (n) parameters a and c, (o) ratio of *c/a* and cell volume from 25 to 550 °C. (p) XRD pattern of cycled cathodes under nitrogen from 25 to 600 °C, and its enlarged area of (q)18.4–19.0°, (r) 43.8–45.0°; The corresponding (s) parameters a and c, (t) ratio of *c/a* and cell volume from 25 to 500 °C.

The XRD measurements were carried out to understand the structure changes of materials after thermal treatment. Figure 3a shows the corresponding XRD results of the fresh cathode, heated under argon, where samples from 250 to 550 °C exhibit a typical diffraction pattern of hexagonal *α*‐NaFeO_2_ layer structure (*R‐3 m* space group), same as the pristine materials (at 25 °C). The 006/102 and 108/110 doublets separate clearly from 250 to 500 °C, suggesting ordered layer structure, but at 550 °C, the spacing between 006/102 and 108/110 doublets decrease and even tend to merge, which indicates the degradation of layered structure. The phase degradation at 550 °C can be ascribed to Li/O release and reduction of transition metal driven by inert atmosphere (no oxygen) and high temperature.^[46]^ Furthermore, a more significant phase degradation occurs at a temperature of 600 °C. During this process, the layered structure undergoes a transformation into a phase consisting of the rock‐salt phase with a cubic structure (*Me*O, *Me*=Ni, Co, *Fm‐3 m* space group), along with the formation of a nickel or cobalt metal phase also exhibiting a cubic structure (*Fm‐3 m* space group). Manganese would be present in the form of Mn_2_O_3_, as some of its peaks coincide with the rock‐salt phase of NiO and CoO, while no peaks corresponding to Mn_3_O_4_ and MnO are observed. This phase transformation is associated with carbothermal reduction triggered by the conductive carbon additive and the decomposition products of PVDF.^[47]^

Additionally, the expanded regions of the XRD patterns (Figure 3b‐c) were analyzed to observe and compare the slight variations in the layer structure. The positions of 003 and 104 reflections between 250 and 550 °C constantly fluctuate in a small range. In general, the shift of 003 reflections signifies variations in the lattice parameter *c*, while the displacement of 104 reflections indicates changes in the lattice parameter *a*. In order to further confirm the structural variation, the cell parameters were obtained through Rietveld refinement (Figure 3d‐e and Table S1). Parameter *a* and the cell volume consistently increase with the temperature rising, while parameter *c* exhibits an irregular variation. These changes could be ascribed to many reasons, including the oxygen release, the reduction of TM, and the corrosion by HF after the decomposition of PVDF. Nevertheless, accurately illustrating the precise mechanism behind the parameter variation remains challenging.

It is crucial to emphasize that the *c/a* ratio offers valuable insights into the structural ordering of phases. In the case of a perfect cubic rock‐salt phase, exemplified by NiO, the cubic lattice can be represented in a hexagonal lattice with parameters $a_{H}$ =$a_{c}/\sqrt{2}$ and $c_{H}$ =${2\sqrt{3}a}_{c}$ resulting in a *c/a* ratio of $2\sqrt{6}=$ 4.899. In *R‐3 m* layered transition‐metal oxides, the *c/a* ratio is expected to exceed 4.899 due to weak interlayer forces, causing a subtle separation of transition‐metal layers along the *c*‐axis. Literatures on well‐ordered layer structures of NMC622 materials suggest a *c/a* ratio of approximately 4.96.[^46^, ^49^] In our study, the initial and fresh NMC622 material exhibits a *c/a* ratio of 4.958, assumed to be the optimal value for our NMC622 materials, indicating an ideal layer structure ordering. Consequently, the assessment of structural degradation after thermal treatment involves comparing the calculated *c/a* ratio with the reference value of 4.958. As shown in Figure 1e, the ratios under argon gradually decrease as the temperature rises, which is gradually deviating from the reference value of 4.958, indicating that the layer structure undergoes continuous structural degradation.

In cycled cathodes, the layered hexagonal structure is preserved only within the temperature range of 250 to 500 °C (Figure 1f). At 550 °C, it transforms into a rock‐salt phase, with a small presence of nickel or cobalt metal phase (Figure S4). At 600 °C, this transformation becomes more pronounced, resulting in a higher proportion of the nickel or cobalt metal phase. The significant degradation of the layered structure occurs at 500 °C, which is 50 °C lower than in the fresh cathode, according to the separation of 006/102 and 108/110 doublets. In addition, it is observed that the 003 and 104 reflections exhibit a larger angle shift in comparison to the fresh cathodes (Figure 3g‐h). This observation can be further explained by considering the variations in the cell parameters. The calculated cell parameters show that, at 500 °C, the lattice parameters *a*, *c*, and cell volume of the obtained materials expand on 0.600 %, 0.203 %, and 1.412 %, respectively, compared to the original materials, which are higher than the 0.112 %, 0.003 % and 0.243 % of fresh cathodes, treated under argon at the same temperature, indicating a more obvious structure change in cycled cathode. Additionally, the *c/a* ratio for a fresh cathode at 500 °C is 4.955, while for cycled cathode at the same temperature, it is 4.936 (Figure 3j). These findings indicate that the degradation of the layer structure upon thermal treatment is more pronounced in cycled cathodes, primarily due to the degraded structural stability resulting from the cycling process. Interestingly, the ratio of *c/a* exhibits an increase at 400 and 450 °C, approaching the value of 4.958. We would attribute this to the expansion of the material structure along the *c*‐axis, given that parameter *a* shows no significant change (Figure 3i). The pronounced expansion of parameter *c* may mainly result from the release of Li/O in the cycled material, which, to some extent, influences the cation arrangement and exacerbates the interlayer repulsion.

#### 2.2.2 Cathodes under Nitrogen

Figure 3k‐t presents the XRD results of the samples after thermal treatment under a nitrogen atmosphere, which exhibit similar behavior to the results obtained under argon. In the case of fresh cathodes, the phases observed in the temperature range of 250 to 550 °C can be indexed to the layer structure (Figure 3k). However, at 550 °C, a noticeable degradation of the layer structure is observed, as evidenced by the separation of the 006/102 and 108/110 doublets. At 600 °C, the layer structure transforms to the rock‐salt and nickel or cobalt metal phases. The shift of 003 and 104 reflections remains small from 250 to 500 °C (Figure 3l‐m). The obtained cell parameters reveal a consistent increase in parameter *a* and cell volume (Figure 3n‐o and Table S3), while the variation of parameter *c* does not exhibit a clear regularity as the temperature increases. The continuous decrease of the ratio of *c/a* indicates a progressively severe degradation of layer structure (Figure 3o). In the case of cycled cathodes, from 250 to 450 °C, the material maintains a well‐ordered layer structure (Figure 3p). However, a significant degradation occurs at 500 °C, leading to transformation into a rock‐salt phase and nickel or cobalt metal phase (Figure S5) at 550 °C. Further heating to 600 °C results in an increased proportion of nickel or cobalt metal phase. The shift of 003 and 104 reflections towards higher diffraction angles was observed in the cycled cathodes compared to the fresh cathodes (Figure 3q‐r). The obtained cell parameter shows that parameter *a* continuously increases with the temperature increase, while parameter *c* and the cell volume constantly rise from 250 to 450 °C but decrease at 500 °C (Figure 3s‐t and Table S4). The ratio of *c/a* increases at 400 and 450 °C, consistent with the results under argon, and then decreases to 4.948 at 500 °C.

#### 2.2.3 Cathodes under Hydrogen

**Figure 4:**
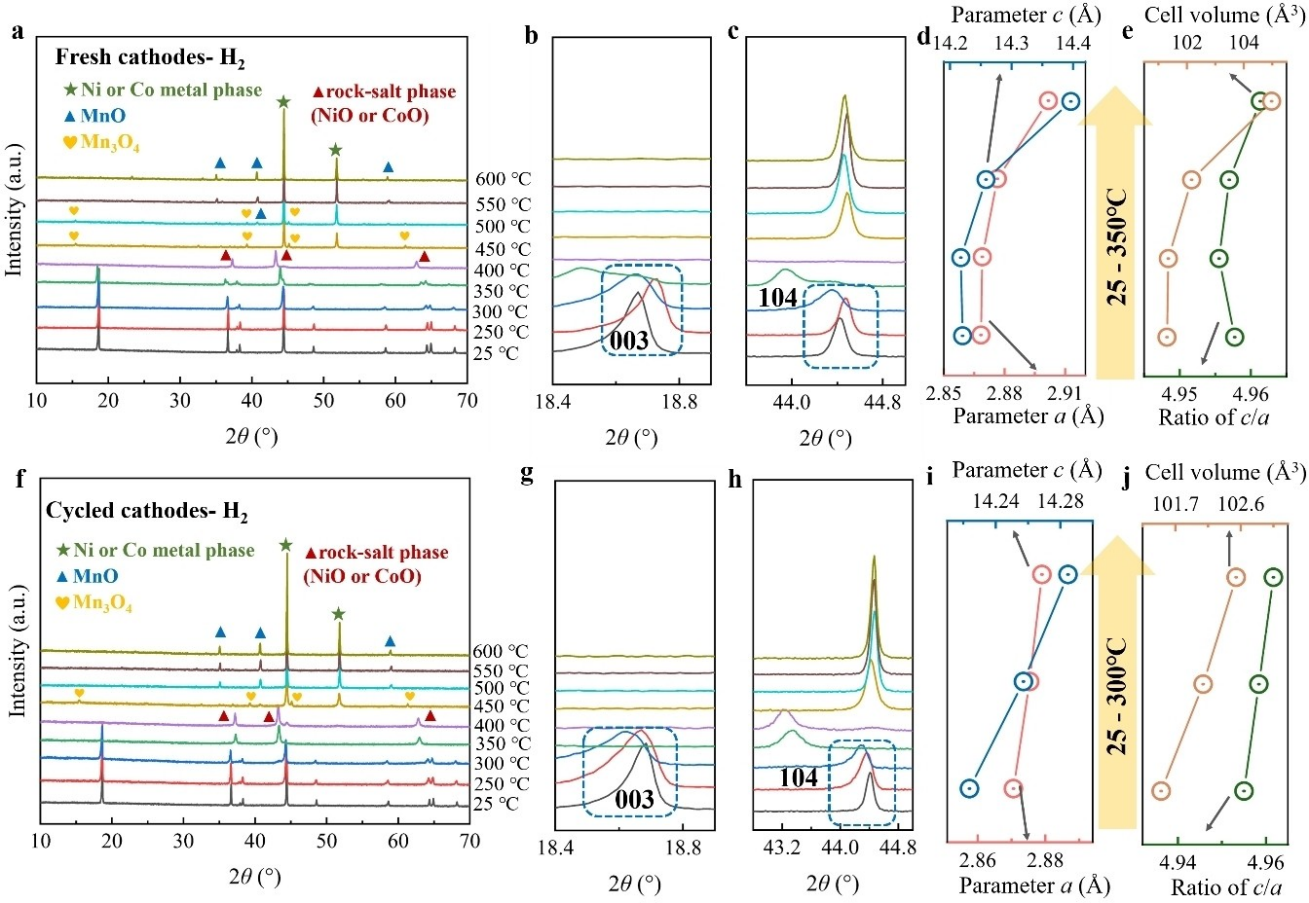
***Cathodes under hydrogen***. (a) XRD pattern of fresh cathodes under hydrogen from 25 to 600 °C, and its area of (b)18.4–19.0°, (c) 43.8–45.0°; The corresponding (d) parameters *a* and *c*, (e) ratio of *c/a* and cell volume from 25 to 350 °C. (f) XRD pattern of cycled cathodes under hydrogen from 25 to 600 °C, and its enlarged area of (g)18.4–19.0°, (h) 42.8–45.0°; The corresponding (i) parameters *a* and *c*, (j) ratio of *c/a* and cell volume from 25 to 300 °C.

Figure 4a–j presents the XRD results of the samples after thermal treatment under a hydrogen atmosphere. Heated under reducing atmosphere, the fresh cathode materials exhibit a hexagonal *α*‐NaFeO_2_ layer structure (*R‐3 m* space group) within the temperature range of 250 to 350 °C. Notably, the 006/102 and 108/110 doublets, after reaching 300 °C, exhibit a tendency to merge, indicating a characteristic sign of structural degradation (Figure 4a). By the time the temperature reaches 400 °C, the layered structure undergoes a transformation into a phase characterized by the rock‐salt phase with a cubic structure (*Me*O, *Me*=Ni, Co). Simultaneously, a metal phase of nickel and cobalt emerges, also showcasing a cubic structure (*Fm‐3 m* space group), resembling the phase observed in fresh cathodes under argon and nitrogen at 550 °C. At 450 °C, the phase initially observed at 400 °C undergoes additional transformation. The nickel and cobalt oxides tend to fully convert into nickel or cobalt metal phases, resulting in aluminum becoming brittle and ultimately breaking into powder during the sonication process. This transformation is also accompanied by the formation of a manganese oxide (Mn_3_O_4_) phase. Beyond 500 °C, Mn_3_O_4_ transforms into MnO phase. Therefore, under a hydrogen atmosphere, the materials undergo a more substantial structural decomposition compared to argon and nitrogen. According to the separation experiments, the fresh cathode achieves 100 % separation efficiency at 400 °C under hydrogen, whereas the same efficiency is attained at 500 °C under argon and nitrogen. The lower temperature requirement under hydrogen can be attributed to the significant phase transformation of transition metal oxides. This transformation contributes to the disruption of bonds between aluminum foil and active material, thereby facilitating the separation process. Additionally, Figure 4b‐c depict expanded sections of the XRD patterns and the calculated cell parameters in the temperature range of 25 to 350 °C. It is evident that, while maintaining the layer structure, the material exhibits an increase in parameters *a*, *c*, and cell volume with rising temperature (Figure 4d‐e and Table S5). The *c/a* ratio also follows a similar pattern. This increase is attributed to the over‐expansion of the lattice along the c‐axis, stemming from reduction reactions, layer structure distortion, and phase transformations induced by hydrogen.

In cycled cathodes, similar to those cycled under argon and nitrogen, the phase transformation occurs at a lower temperature when compared to fresh cathodes. As depicted in Figure 4f, the layered hexagonal structure is maintained only at 250 and 300 °C. Beyond this range, a phase transitions to the rock‐salt phase occurs at 350 °C. Moreover, at 400 °C, a subtle metal phase of nickel or cobalt emerges alongside the rock‐salt phase. Similar to fresh cathodes under hydrogen, the Mn_3_O_4_ phase appears from 450 °C and the nickel and cobalt oxides totally transform into metal phase. Beyond 500 °C, MnO and metal phase coexist in the obtained materials. In the separation experiment, the cycled cathodes exhibit an almost 100 % separation efficiency at 400 °C, and have a remarkably higher separation efficiency than fresh cathode under hydrogen at 350 °C. This highlights the significant influence of the phase transformation of transition metal oxides on separation efficiency. Yet, due to the aluminum corrosion during cycling and exposure to air after disassembly, the total separation could become more difficult compared to fresh cathodes. Furthermore, the expanded segments of the XRD patterns and the calculated cell parameters within the temperature range of 25 to 300 °C are shown in Figure 4g‐j and Table S6. These variations correspond to the observed regularities seen in the fresh cathode.

#### 2.2.4 Cathodes under Air

**Figure 5:**
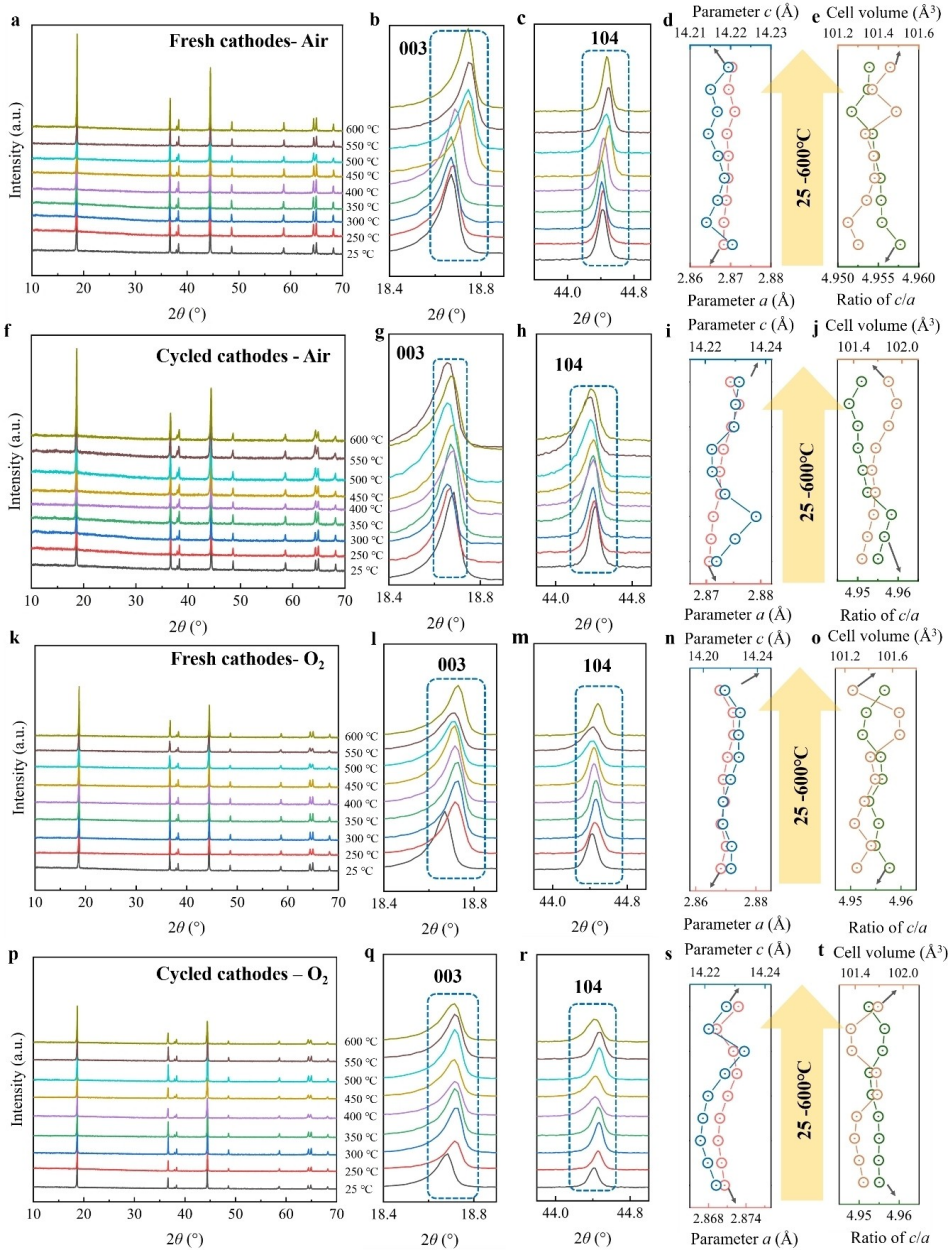
***Cathodes under air***. (a) XRD patterns of fresh cathodes under air from 25 to 600 °C, and its enlarged area of (b)18.4–19.0°, (c) 43.8–45.0°; The corresponding (d) parameters *a* and *c*, and (e) ratio of *c/a* and cell volume. (f) XRD patterns of cycled cathodes under air from 25 to 600 °C, and its enlarged area of (g)18.4–19.0°, (h) 43.8–45.0°; The corresponding (i) parameters *a* and *c*, and (j) ratio of *c/a* and cell volume from 25 to 600 °C. ***Cathodes under oxygen***. (k) XRD patterns of fresh cathodes under oxygen from 25 to 600 °C, and its enlarged area of (l)18.4–19.0°, (m) 43.8–45.0°; The corresponding (n) parameters *a* and *c*, and (o) ratio of *c/a* and cell volume. (p) XRD patterns of cycled cathodes under oxygen from 25 to 600 °C, and its enlarged area of (q)18.4–19.0°, (r) 43.8–45.0°; The corresponding (s) parameters *a* and *c*, and (t) ratio of *c/a* and cell volume from 25 to 600 °C.

***Cathodes under air***. Figure 5a‐j exhibits the XRD results of the samples treated in air. Fresh cathodes keep the layer structure from 250 to 600 °C. The spacing of 006/102 and 108/110 doublets indicate an ordered layer structure for all samples. The shift of 003 and 104 reflections reflects the changes in layer structure (Figure 5b‐c), which can be further explained by the obtained cell parameters (Figure 5d‐e and Table S7). As we see, the variation of parameters *a*, *c*, and cell volume is small. The ratio of *c/a* decreases continuously with increasing temperature, but it remains higher than 4.95. Besides, these values are closer to 4.958 compared to results obtained under argon and nitrogen atmospheres at the same temperatures. These results suggest that the air atmosphere favors maintaining the layer structure stability of materials during thermal treatment. The structural stability under air could benefit from the existence of oxygen, which contributes to inhibiting the Li/O releasing from the bulk phase, thus enabling a much slighter structure degradation under air.

Similarly, for the cycled cathodes, the observed phases from 250 to 600 °C retain the hexagonal layer structure, and the spacing of the 006/102 and 108/110 doublets indicates a well‐maintained layer structure (Figure 5f). The shift of 003 and 104 reflections and the variation of cell parameters are displayed in Figure 5g‐j. The position of 104 reflections continuously shifts towards a lower 2 Theta angle at temperatures from 250 to 550 °C and then towards a higher angle at 600 °C, which is consistent with the variation of parameter *a* (Figure 5i and Table S8). The shift of the 003 reflections and the changes in parameter *c* and cell volume exhibit a more irregular pattern in the cycled cathodes. Meanwhile, the *c/a* ratio shows a decreasing trend with increasing temperature, and these ratios are all lower than those of the fresh cathodes under the same conditions (Figure 5j). This indicates that the cycled cathodes experience more pronounced structure degradation, which is consistent with the results obtained under both argon and nitrogen atmospheres.

#### 2.2.5 Cathodes under Oxygen

Figure 5k‐t exhibits the XRD results of the samples treated on oxygen. Similar to the finding under air, fresh cathodes retain the layered structure, exhibiting good structural ordering from 250 to 600 °C (Figure 5k). A comparison with the air atmosphere reveals that the 003 and 104 reflections under oxygen experience a smaller shift (Figure 5l‐m). This corresponds to a slight alteration in parameters *a*, *c*, and cell volume, as illustrated in Figure 5n‐o and Table S9. Furthermore, the *c/a* ratio consistently remains above 4.95, with values approaching 4.958 in contrast to the air atmosphere. These results suggest a superior degree of structural ordering under oxygen atmosphere. For cycled cathodes, the materials maintain a well‐ordered layer structure from 250 to 600 °C (Figure 5p), accompanied by a smaller shift in the 003 and 104 reflections (Figure 5q‐r) and slight variations in parameters *a*, *c*, and cell volume (Figure 5s‐t and Table S10). The *c/a* ratios in this temperature range hover around 4.955. These results indicate that the presence of an oxygen atmosphere can effectively impede structural degradation.

Finally, the rate of variation of cell parameters and *c/a* ratio for fresh and cycled cathodes under temperatures that can achieve 100 % separation efficiency while maintaining the layered structure is summarized in Table 1. The information presented in the Table 1 indicates that both air and oxygen atmosphere contribute to achieving 100 % separation efficiency while minimizing structural degradation. Specifically, under an air atmosphere, the suitable temperature range is determined to be from 400 to 450 °C. Conversely, under an oxygen atmosphere, the recommended temperature range shifts slightly lower, spanning from 350 to 450 °C.

**Table 1:** Variation rate of cell parameters for fresh and cycled cathodes compared with original materials, while the ratio of *c/a* is compared with reference value of 4.958. The values highlighted in bold indicate the suitable conditions for thermal treatment, ensuring the highest separation efficiency while minimizing structural degradation.

	Atmosphere	Temperature (°C)	Variation rate of cell parameters (%)			*c/a* (%)
			*a*	*c*	volume	
Fresh cathodes	Argon	500	0.112	−0.003	0.243	−0.061
		550	0.313	0.134	0.783	−0.443
	Nitrogen	500	0.013	−0.076	−0.030	−0.100
		550	0.447	0.182	1.101	−0.262
	Hydrogen	400	/	/	/	/
	Air	**400**	**0.039**	**−0.025**	**0.075**	**−0.061**
		**450**	**0.028**	**−0.042**	**0.035**	**−0.081**
		500	0.096	−0.026	0.186	−0.121
		550	0.043	−0.038	0.068	−0.081
		600	0.071	−0.007	0.156	−0.081
	Oxygen	**350**	**0.041**	**−0.041**	**0.063**	**−0.081**
		**400**	**0.028**	**−0.002**	**0.152**	**−0.040**
		**450**	**0.076**	**0.041**	**0.113**	**−0.040**
		500	0.147	0.039	0.355	−0.101
		550	0.141	0.048	0.351	−0.101
		600	**−**0.012	**−**0.032	**−**0.035	−0.020
Cycled cathodes	Argon	500	0.600	0.206	1.412	−0.444
	Nitrogen	500	0.170	0.032	0.371	−0.202
	Hydrogen	400	/	/	/	/
	Air	**400**	**0.066**	**−0.010**	**0.121**	**−0.141**
		**450**	**0.090**	**−0.011**	**0.167**	**−0.161**
		500	0.142	0.040	0.324	−0.161
		550	0.189	0.044	0.423	−0.202
		600	0.135	0.053	0.323	−0.141
	Oxygen	**350**	**−0.020**	**−0.045**	**−0.055**	**−0.088**
		**400**	**−0.033**	**−0.006**	**0.034**	**−0.035**
		**450**	**0.015**	**0.037**	**−0.006**	**−0.041**
		500	0.086	0.036	0.237	−0.114
		550	0.080	0.045	0.233	−0.098
		600	−0.073	−0.035	−0.153	−0.025

#### 2.2.6 Potential Application of Thermal Treatment for Cycled Cathodes

From the perspective of direct recycling, treating cycled cathodes in an inert or reducing atmosphere is generally undesirable due to severe phase degradation, which significantly challenges the regeneration of these materials. However, this phase degradation may benefit hydrometallurgical processes. Typically accompanied by the release of lithium and oxygen, the degradation facilitates the selective extraction of lithium in water‐leaching processes and enhances the dissolution of transition metals in acid‐leaching processes due to their reduced oxidation states.

Conversely, a direct recycling process may benefit from treating cycled cathodes in the air or oxygen atmospheres. Although the separation efficiency of the cycled cathode at 350 °C in oxygen is noticeably higher than in air, it does not reach 100 %. This outcome suggests that both air and oxygen atmospheres exhibit similar temperature thresholds for maximizing separation efficiency. Given this, an air atmosphere could be more economically favorable. From an energy consumption standpoint, 400 °C is preferable to higher temperatures. However, carbon additives in cycled cathodes typically decompose completely at temperatures approaching 600 °C, even under air.^[36]^ Therefore, it may be necessary to increase the temperature or extend the holding time to reduce the proportion of these carbon additives in the recovered active material.

Impurities in the obtained active material are a significant concern. There are two primary sources of impurities in this process. One is from the aluminum foil, primarily due to the erosion caused by HF released during the decomposition of PVDF. This phenomenon resulting in aluminum powder impurities is more pronounced at larger scales of cathode processing due to the more intense release of HF. The ICP‐OES and TECG results shown in Table S11 demonstrate the elemental proportions after larger‐scale thermal treatment of cycled cathodes (~30 g) at 400, 450, and 500 °C. The weight percent of aluminum impurity varies between 0.3 to 0.5 *wt %*, likely due to its non‐uniform distribution. While we consider this level of aluminum impurity acceptable, its impact on the regeneration process of cathode materials requires further investigation.

Another source of impurities is the decomposed products from CEI, which introduce a significant amount of lithium compounds on the surface of the active materials. This is evidenced by the differing stoichiometries of the cycled material of 450 °C with and without water‐ethanol washing: Li_1.004_Ni_0.601_Mn_0.200_Co_0.199_O_1.921_ and Li_0.856_Ni_0.601_Mn_0.201_Co_0.198_O_1.905_ respectively (Table S12). A notable decrease in lithium content is observed after washing, indicating that while lithium‐containing impurities may potentially serve as a supplementary lithium source during regeneration, their precise effects also require further exploration.

### 2.3 Structural Stability Investigations of Anodes

#### 2.3.1 Anodes under Argon

**Figure 6:**
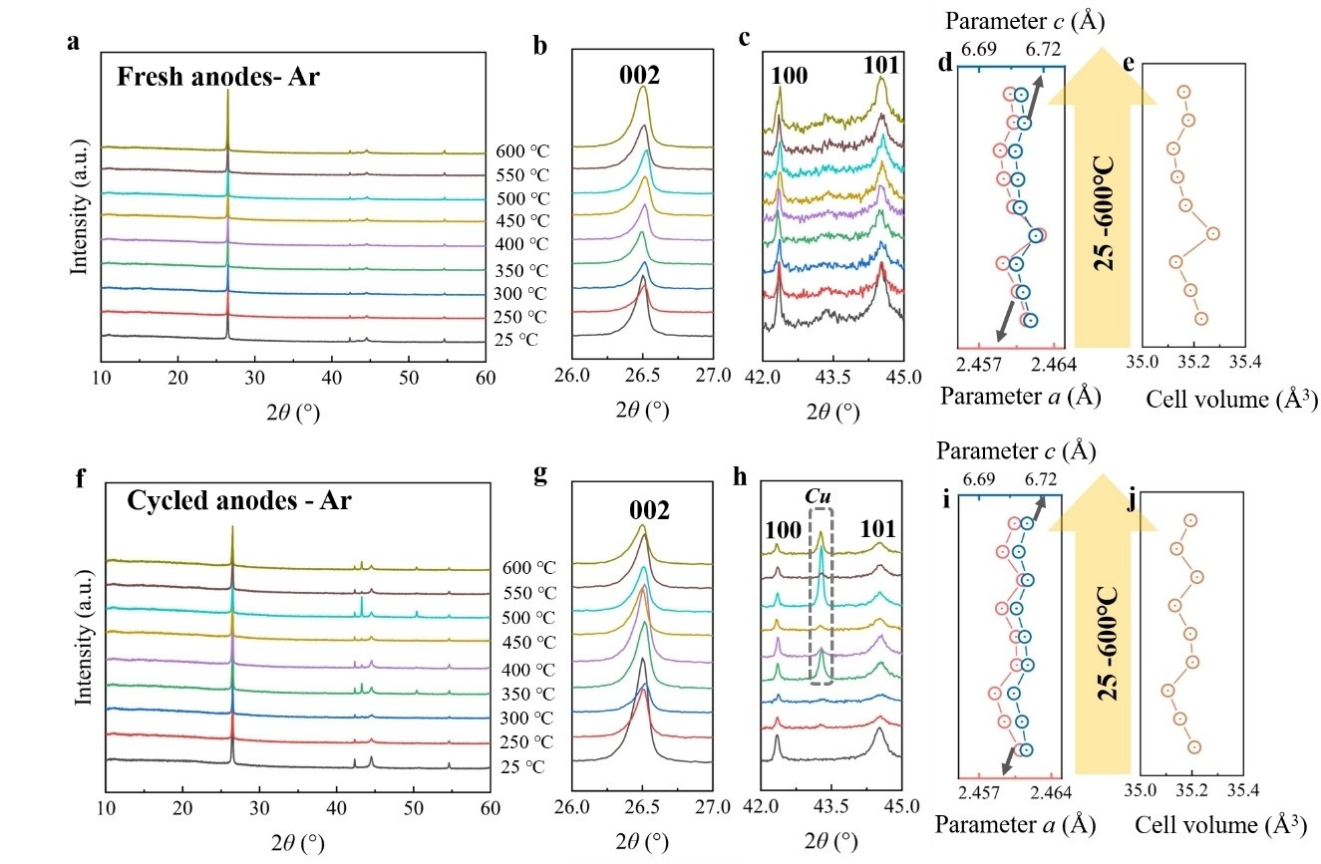
***Anodes under argon***. (a) XRD patterns of fresh anodes under argon from 25 to 600 °C, and its enlarged area of (b) 26.0–27.0°, (c) 42.0–45.0°; The corresponding (d) parameters *a* and *c*, and (e) cell volume. (f) XRD pattern of cycled anodes under argon from 25 to 600 °C, and its enlarged area of (g) 26.0–27.0°, (h) 42.0–45.0°; The corresponding (i) parameters *a* and *c*, and (j) cell volume.

The XRD results of the fresh and cycled anodes after thermal treatment under argon atmosphere are displayed in Figure 6a‐j. The materials obtained from the fresh anodes within the temperature range of 250 to 600 °C can be indexed in a hexagonal layer structure with the *P63/mmc* space group. The shift of 002 reflections represents the change of interlayer distance and lattice parameter *c*, while the shift of 100 and 101 reflections corresponds to the variation of parameter *a*. As shown in Figure 6b c, there is only a small shift for the 002, 100, and 101 reflections from 250 to 600 °C, corresponding to a slight variation of cell parameters (Figure 6de and Table S13), which indicates good structure stability of graphite at this atmosphere and temperature range. The obtained materials from 250 to 600 °C for the cycled anodes also maintain the layer structure. The observed slight shift in reflection position and the variation in cell parameters (Figure 6g‐j and Table S14) suggest that the cycled materials also possess good structural stability when exposed to high temperatures. Notably, reflections attributed to copper are observed in the temperature range of 350 to 600 °C. Their presence can be attributed to chemical changes occurring at the interface contact between graphite and copper during thermal treatment. These changes result in the inclusion of copper impurities in the graphite after the scraping process.

#### 2.3.2 Anodes under Nitrogen

**Figure 7:**
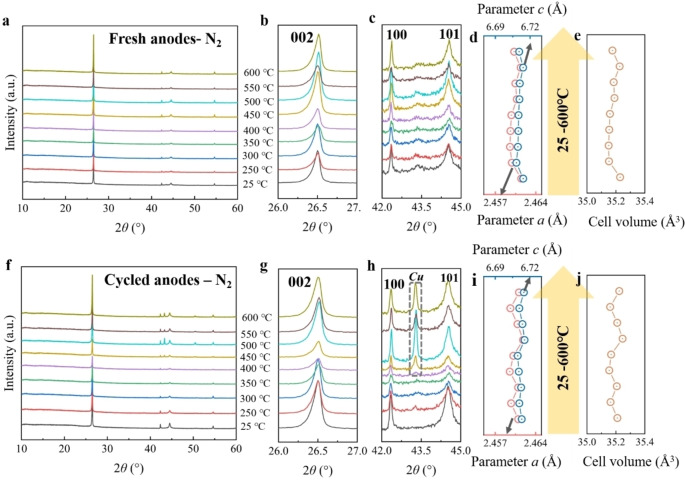
***Anodes under nitrogen***. (a) XRD patterns of fresh anodes under nitrogen from 25 to 600 °C, and its enlarged area of (b) 26.0–27.0°, (c) 42.0–45.0°; The corresponding (d) parameters *a* and *c*, and (e) cell volume. (f) XRD spectrum of cycled anodes under nitrogen from 25 to 600 °C, and its enlarged pattern of (g) 26.0–27.0°, (h) 42.0–45.0°; The corresponding (i) parameters *a* and *c*, and (j) cell volume.

Figure 7a‐j shows the XRD results of the fresh and cycled anodes after thermal treatment under a nitrogen atmosphere. For the fresh cathodes, the materials exhibit a layered structure in the temperature range of 250 to 600 °C, which is similar to the results obtained under an argon atmosphere. The shifts of 002, 100, and 101 reflections at these temperatures are not noticeable (Figure 7b‐c). Correspondingly, the cell parameters have only slight variation (Figure 7d‐e and Table S15), which implies good structure stability of graphite under this temperature range. For the cycled cathodes, the materials obtained from 250 to 600 °C continue to maintain a layered structure. The shifts of 002, 100, and 101 reflections are also very minor (Figure 7g‐h), corresponding to the slight fluctuation of the cell parameters (Figure 7i‐j and Table S16), indicating that the thermal treatment at this temperature range does not have a significant impact on the graphite structure. Yet, the distinct reflections of copper are also observed from 400 to 600 °C, resembling the results observed in cycled anodes under argon atmosphere.

#### 2.3.3 Anodes under Hydrogen

**Figure 8:**
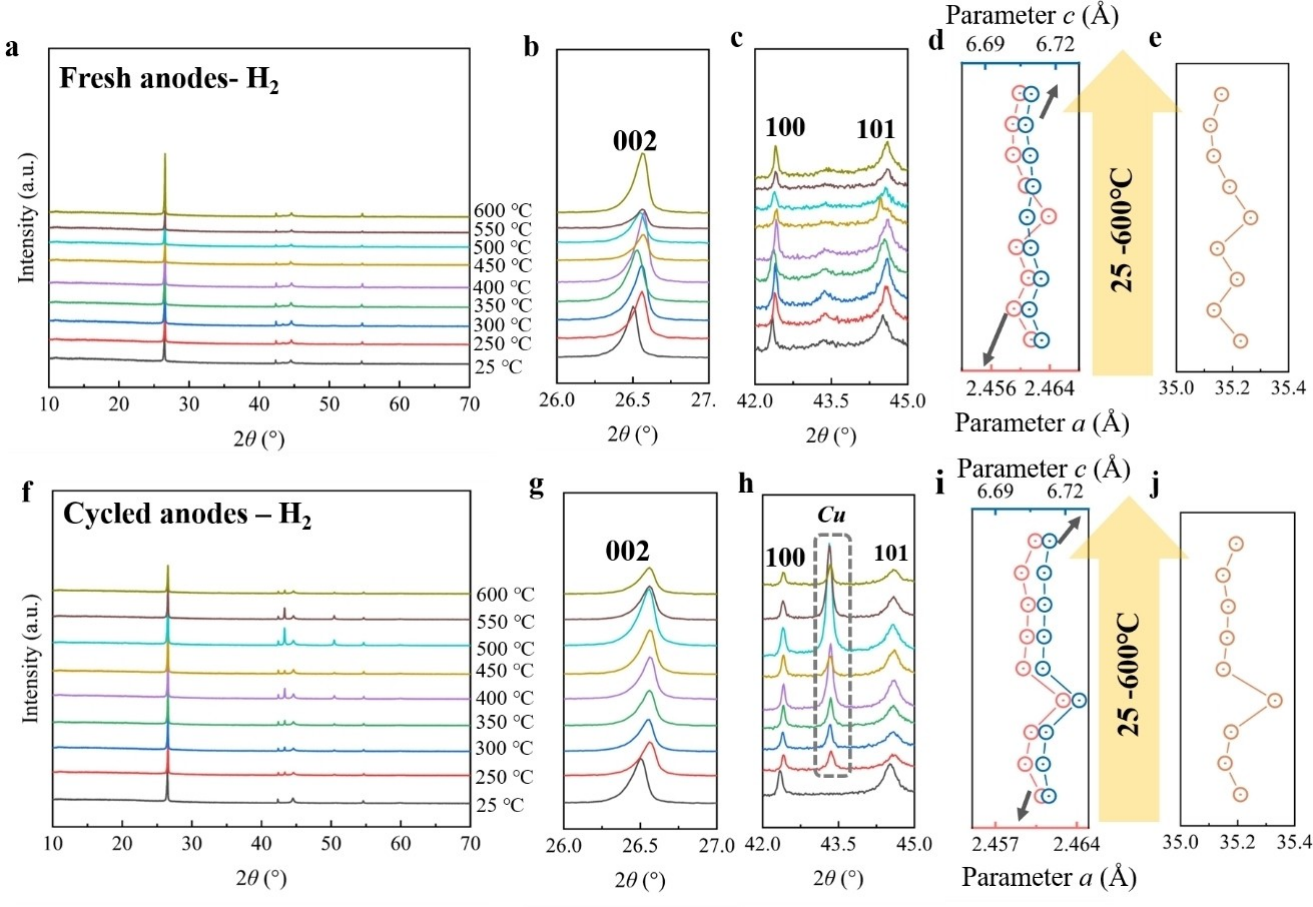
***Anodes under hydrogen***. (a) XRD pattern of fresh anodes under air from 25 to 600 °C, and its partial enlarged area of (b) 26.0–27.0°, (c) 34.0–46.0°, (d) 50–70°. (e) XRD pattern of cycled anodes under air from 25 to 600 °C, and its partial enlarged area of (f) 26.0–27.0°, (g) 34.0–46.0°, (h) 50–70°.

Figure 8a‐j shows the XRD results of the fresh and cycled anodes after thermal treatment under a hydrogen atmosphere. In the case of the fresh anodes, the materials exhibit a layered structure within the temperature range of 250 to 600 °C, mirroring the outcomes observed under argon and nitrogen atmospheres. Notably, there are no discernible shifts in the 002, 100, and 101 reflections at these temperatures (Figure 7b c). Correspondingly, there is only a slight variation in cell parameters (Figure 7d‐e and Table S17), indicating the robust structural stability of graphite within this temperature range. For the cycled anodes, the materials maintained a layered structure within the 250 to 600 °C temperature range. The shifts in 002, 100, and 101 reflections are also minimal (Figure 7g‐h), corresponding to slight fluctuations in cell parameters (Figure 7i‐j and Table S18). This implies, under a hydrogen atmosphere, that thermal treatment within this temperature range has a negligible impact on the graphite structure, akin to the effects observed under argon and nitrogen atmospheres. Besides, more distinct reflections of copper are observed from 400 to 600 °C, resembling the results observed in cycled anodes under argon and nitrogen atmosphere.

#### 2.3.4 Anodes under Air

**Figure 9:**
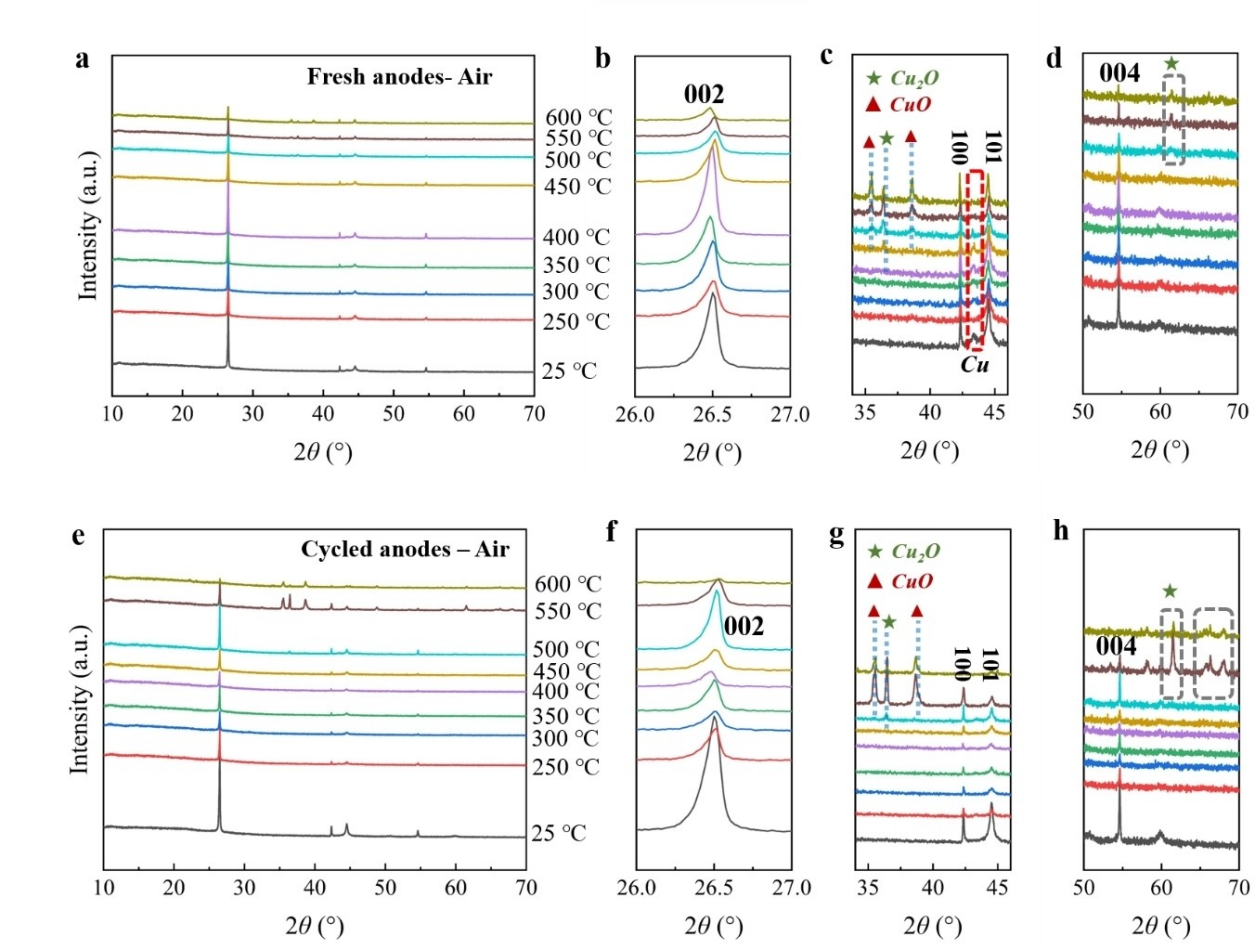
***Anodes under air***. (a) XRD pattern of fresh anodes under air from 25 to 600 °C, and its partial enlarged area of (b) 26.0–27.0°, (c) 34.0–46.0°, (d) 50–70°. (e) XRD pattern of cycled anodes under air from 25 to 600 °C, and its partial enlarged area of (f) 26.0–27.0°, (g) 34.0–46.0°, (h) 50–70°.

Finally, Figure 9a‐h exhibits the XRD results of the cycled and fresh anodes after thermal treatment in air. Note that in this particular section, the calculation of cell parameters is omitted due to the oxidation of graphite and copper and unsatisfactory separation results. Consequently, the study on the cell parameters of graphite is considered less appealing. Nevertheless, the structure variation can still be determined based on shifts and intensity of reflections. Materials of the fresh cathodes maintain the layered structure upon heating from 250 to 600 °C. There is no significant shift of 002, 100, 101, and 004 reflections. The reflection intensity of graphite notably decreases from 450 to 600 °C, indicating the oxidation of graphite. Additionally, certain reflections attributed to CuO and Cu_2_O are observed within the same temperature range, which further confirms the oxidation of copper foil. By combining the XRD analysis with the separation results, it is evident that the oxidation of copper occurs before 250 °C. Notably, before reaching 350 °C, the oxidation of copper is relatively minor and primarily affects the surface. Therefore, even after the decomposition of the binder, the active materials can still be peeled off from the copper foil. As the temperature surpasses 350 °C, the oxidation of copper becomes more pronounced, resulting in the embrittlement of the copper, which poses significant challenges for the separation process.

The layered structure of the cycled anodes remains intact within the temperature range of 250 to 600 °C. At 600 °C, substantial oxidation of graphite is concluded based on the extremely low intensity of graphite reflections. The shift of the 002, 100, 101, and 004 reflections remain within a narrow range from 250 to 550 °C. Additionally, reflections corresponding to CuO and Cu_2_O are observed from 500 to 600 °C, indicating the oxidation of the copper foil.

#### 2.3.5 Potential Application of Thermal Treatment for Cycled Anodes

Thermal treatment of graphite under argon, nitrogen, and hydrogen atmospheres effectively preserves its desired layered structure. However, a significant challenge arises in the separation process, where achieving a complete delamination of active materials is hindered by the deterioration of the interface between graphite and copper foil. This degradation complicates the separation process, as it obstructs the efficient and thorough detachment of graphite. Furthermore, within the temperature range of 350 to 600 °C, incomplete delamination exacerbates the issue, making the simultaneous handling of cycled cathodes and anodes problematic. This leads to an unavoidable mixing of cathode and anode materials.

Given these complications, relying solely on thermal treatment for separating graphite from copper foil is not advisable. Instead, a potentially more efficient approach may involve combining thermal treatment with solvent‐assisted direct recycling techniques. This combination is currently the focus of our ongoing research and will be explored in our upcoming publications.

## 3 Conclusions

In summary, this study provides a comprehensive understanding of the separation effects and structure degradation resulting from the thermal treatment under different atmospheres and temperatures for both fresh and cycled cathodes and anodes.

1. The results reveal that the use of air/oxygen atmosphere allows for achieving 100 % separation efficiency of active cathode materials at a lower temperature compared to argon, nitrogen and hydrogen. Under argon, nitrogen and hydrogen, the layered structure undergoes continuous destruction and transformation as the temperature increases. In contrast, using air and oxygen atmosphere effectively inhibit such transformation and minimizes structure degradation. The structural changes observed under air and oxygen conditions is desirable for direct recycling. Additionally, cycled cathode materials experience more severe phase degradation compared to fresh materials under the same conditions. This is primarily attributed to the degraded structural stability due to cycling.
2. Complete separation of graphite from fresh anodes can be achieved under argon and nitrogen atmosphere at 300 °C, and hydrogen at 350 °C. However, achieving such separation for cycled anodes is challenging due to enhanced interface adhesion between the copper foil and graphite caused by the decomposition of residual compounds on the surface. Nevertheless, graphite exhibits excellent thermal stability under argon, nitrogen and hydrogen, maintaining the hexagonal layer structure from 250 to 600 °C. Yet, air atmosphere induces the oxidation of graphite and copper foil, which affect the separation efficiency and material purity.

## Conflict of Interests

The authors declare no conflict of interest.

4

## Supporting information

As a service to our authors and readers, this journal provides supporting information supplied by the authors. Such materials are peer reviewed and may be re‐organized for online delivery, but are not copy‐edited or typeset. Technical support issues arising from supporting information (other than missing files) should be addressed to the authors.

Supporting Information

## Data Availability

The data that support the findings of this study are available from the corresponding author upon reasonable request.
