# Supplementary material for: Towards Sustainable Direct Recycling: Unraveling Structural Degradation Induced by Thermal Pretreatment of Lithium‐Ion Battery Electrodes
Source: ChemSusChem. 2024 Sep 13;18(1):e202400727. doi: 10.1002/cssc.202400727 (PMC11696215; doi:10.1002/cssc.202400727)
Supplement: Supplementary file 1 — Supporting Information [file CSSC-18-e202400727-s001.pdf]

ChemSusChem

Supporting Information

Towards Sustainable Direct Recycling: Unraveling Structural Degradation Induced by Thermal Pretreatment of Lithium-Ion Battery Electrodes

Shuaiwei Liu, Oleksandr Dolotko, Thomas Bergfeldt, Michael Knapp,* and Helmut Ehrenberg

Supporting Information

Towards Sustainable Direct Recycling: Unraveling Structural Degradation Induced by Thermal Pretreatment of Lithium-Ion Battery Electrodes

Shuaiwei Liu [a,b], Oleksandr Dolotko [a,b], Thomas Bergfeldt [c], Michael Knapp [a,*], Helmut Ehrenberg [a,b]

[a] *Karlsruhe Institute of Technology (KIT), Institute for Applied Materials-Energy Storage Systems (IAM-ESS), Hermann-von-Helmholtz-Platz 1, D-76344 Eggenstein-Leopoldshafen, Karlsruhe, Germany.*

[b] *Helmholtz-Institute Ulm for Electrochemical Energy Storage (HIU), P.O. Box 3640, D-76021 Karlsruhe, Germany.*

[c] *Karlsruhe Institute of Technology (KIT), Institute for Applied Materials-Applied Materials Physics (IAM-AWP), Hermann-von-Helmholtz-Platz 1, D-76344 Eggenstein-Leopoldshafen, Karlsruhe, Germany.*

* **Corresponding author at:** Karlsruhe Institute of Technology (KIT), Institute for Applied Materials-Energy Storage Systems (IAM-ESS), Hermann-von-Helmholtz-Platz 1, D-76344 Eggenstein-Leopoldshafen, Karlsruhe, Germany.

E-mail address: michael.knapp@kit.edu

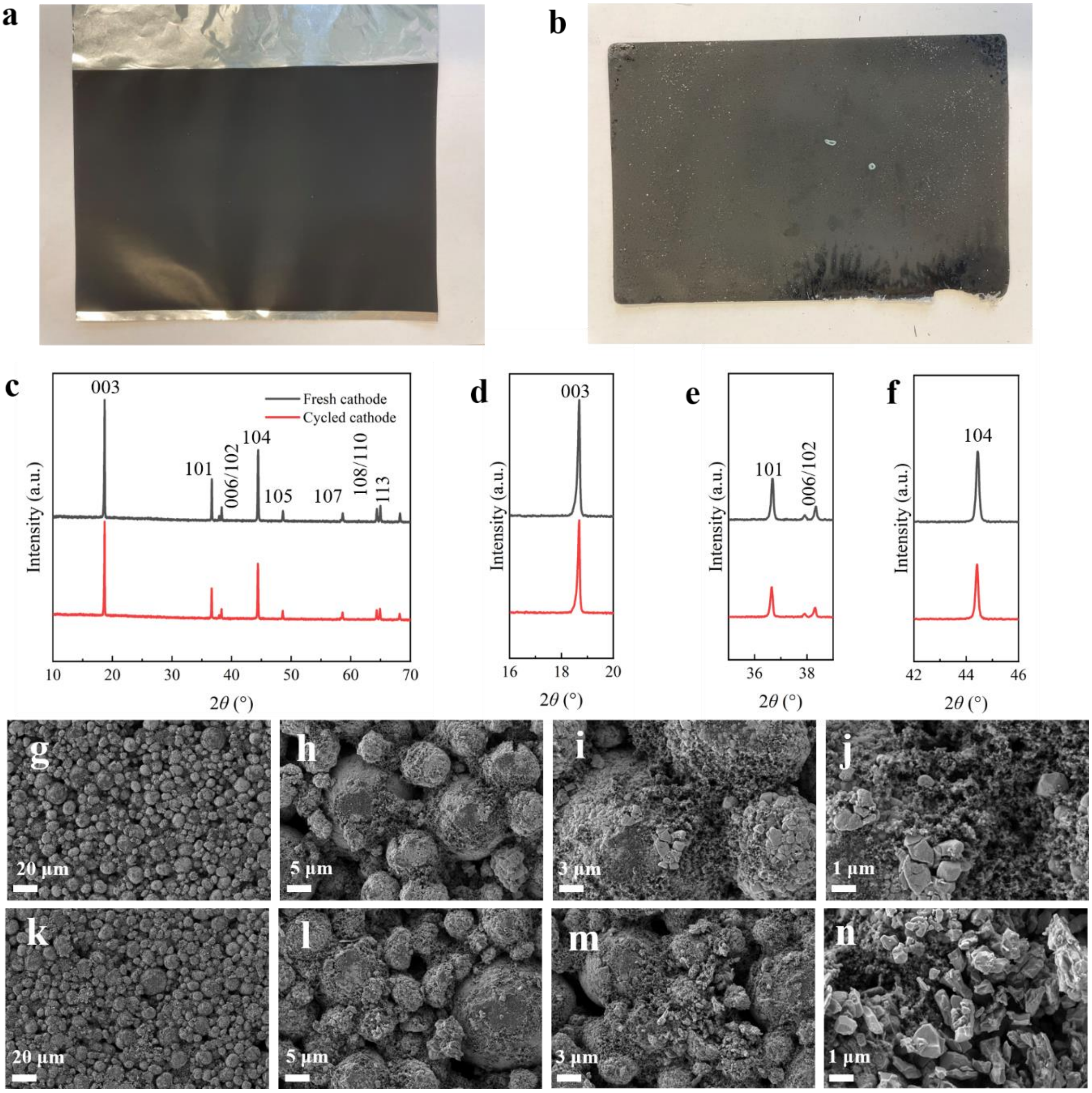

Fig. S1. Appearance of (a) fresh cathode, and (b) cycled cathode; (c) XRD patterns of fresh and cycled cathode, and their enlarged area (d)16.0-20.0°, (e) 37.0-39.0° and (f) 42-46° of 2θ; SEM images of (g-j)-fresh cathode and (k-n)-cycled cathode.

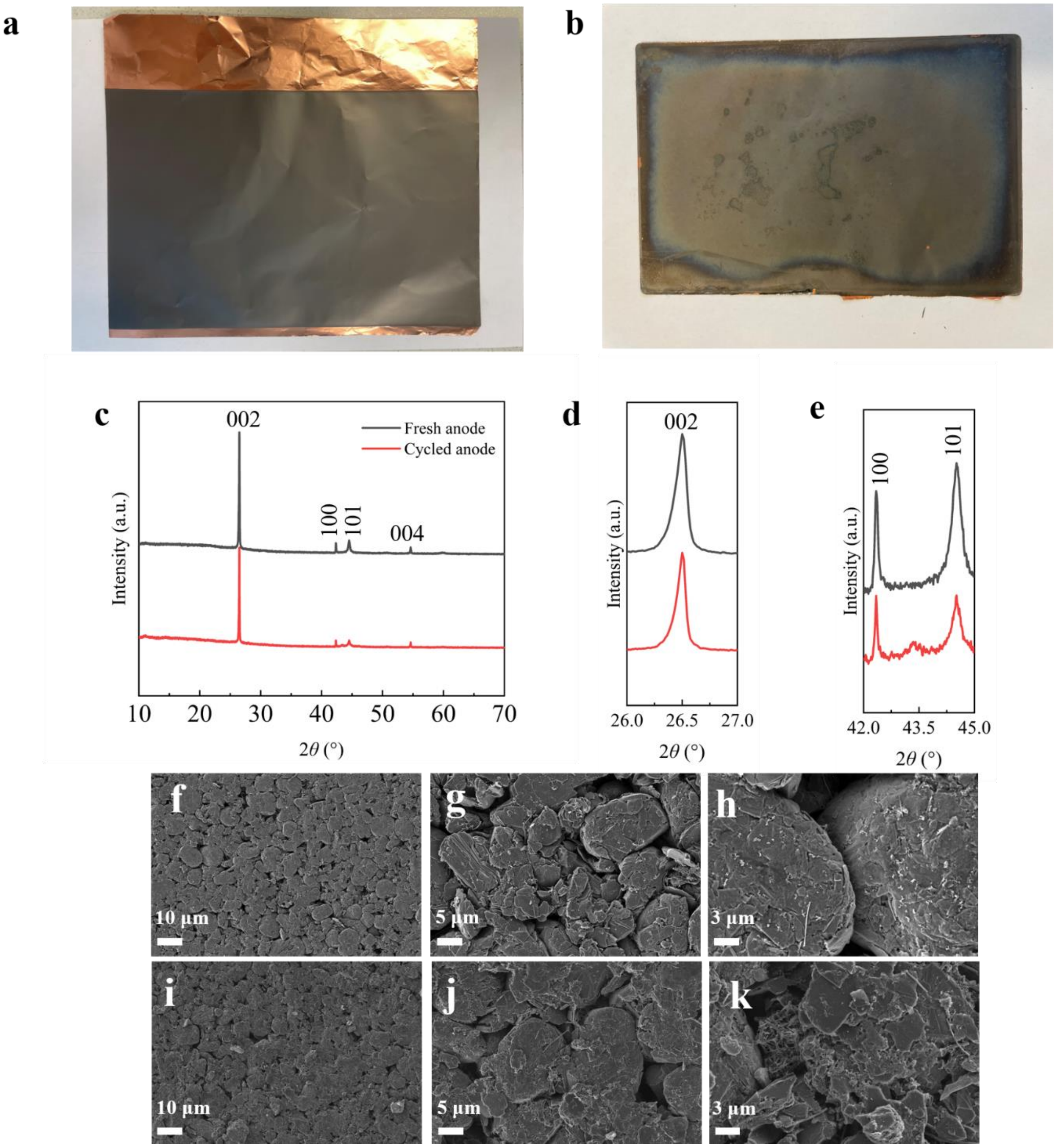

Fig. S2. Appearance of (a) fresh anode, and (b) cycled anode; (c) XRD patterns of fresh and cycled anodes, and their enlarged areas (d)26.0-27.0° and (e) 42.0-45.0° of 2θ; SEM images of (f-h)-fresh anode and (i-k)-cycled anode.

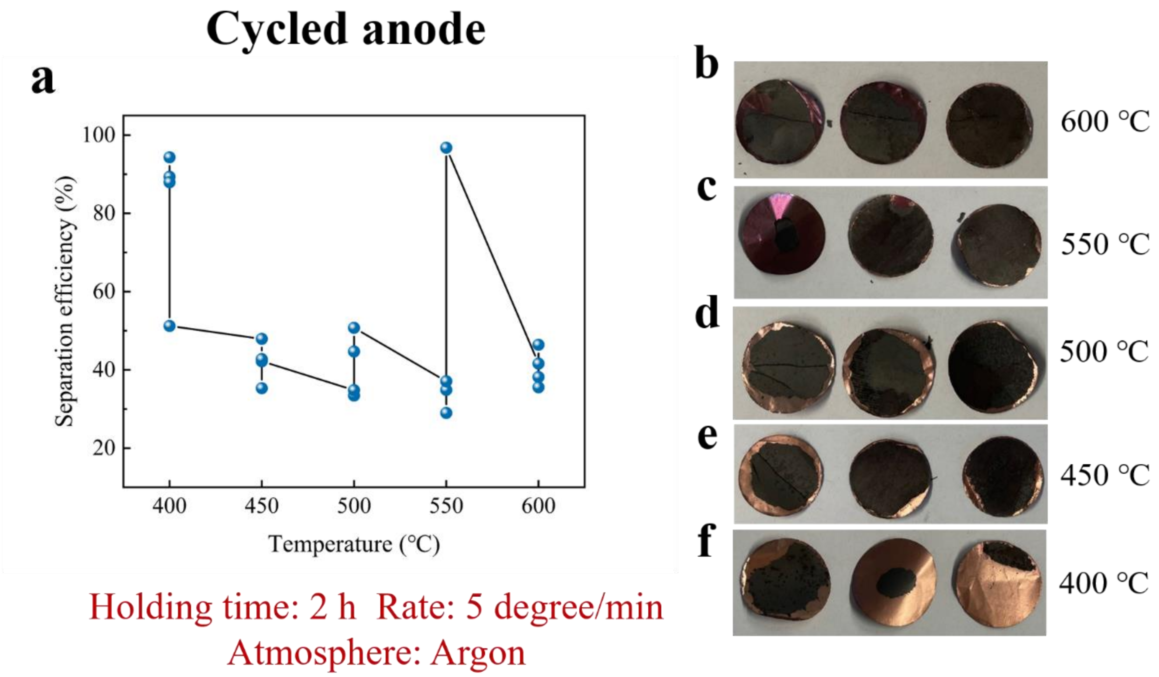

Fig. S3. (a) Separation efficiency of active materials after thermal treatment under argon from 400 to 600 °C for cycled anodes; Electrode state after thermal treatment at (b) 600, (c) 550, (d) 500, (e) 450 and (f) 400 °C.

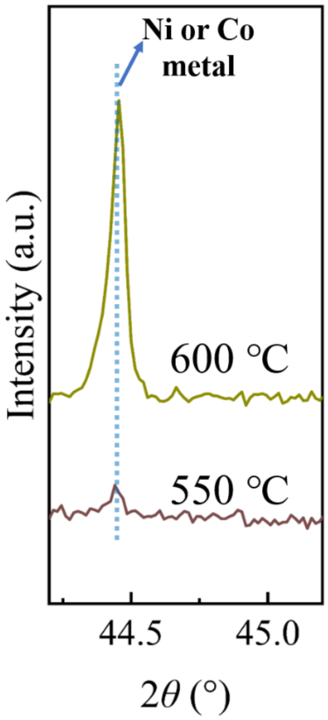

Fig. S4. Enlarged area of XRD pattern of the cycled cathode, heated in an argon atmosphere.

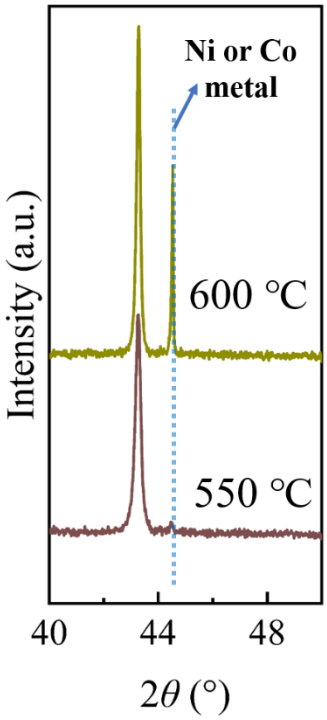

Fig. S5. Enlarged XRD pattern of the cycled cathodes, heated in a nitrogen atmosphere.

Table S1. Cell parameters for the **fresh cathodes** after thermal treatment **under argon**.

The phase from 25 to 550 °C is hexagonal α-$NaFeO_2$ layer structure (*R-3m* space group); *phase-1* is transition metal oxides of cubic structure (*Fm-3m* space group); *phase-2* is nickel or cobalt metal of cubic structure (*Fm-3m* space group). Residuals R_p and R_{wp} do not eliminate the contribution of background fitting but exclude the influence of background noise.

<table>
<tr><th colspan="2">Temperature (°C)</th><th>a (b) (Å)</th><th>c (Å)</th><th>Volume (Å^3)</th><th>c/a</th><th>R_p, %</th><th>R_{wp}, %</th></tr>
<tr><td colspan="2">25</td><td>2.8683(0)</td><td>14.2205(4)</td><td>101.301(3)</td><td>4.958</td><td>2.91</td><td>3.76</td></tr>
<tr><td colspan="2">250</td><td>2.8685(0)</td><td>14.2142(4)</td><td>101.289(3)</td><td>4.955</td><td>3.30</td><td>4.16</td></tr>
<tr><td colspan="2">300</td><td>2.8681(0)</td><td>14.2149(3)</td><td>101.266(3)</td><td>4.956</td><td>3.23</td><td>4.10</td></tr>
<tr><td colspan="2">350</td><td>2.8699(0)</td><td>14.2220(3)</td><td>101.444(3)</td><td>4.956</td><td>3.20</td><td>4.09</td></tr>
<tr><td colspan="2">400</td><td>2.8700(0)</td><td>14.2183(3)</td><td>101.424(3)</td><td>4.954</td><td>3.19</td><td>4.06</td></tr>
<tr><td colspan="2">450</td><td>2.8696(0)</td><td>14.2164(4)</td><td>101.382(4)</td><td>4.954</td><td>3.15</td><td>4.02</td></tr>
<tr><td colspan="2">500</td><td>2.8716(1)</td><td>14.2200(8)</td><td>101.547(5)</td><td>4.952</td><td>3.39</td><td>4.81</td></tr>
<tr><td colspan="2">550</td><td>2.8773(3)</td><td>14.23948(34)</td><td>102.094(27)</td><td>4.949</td><td>4.14</td><td>6.48</td></tr>
<tr><td rowspan="2">600</td><td>phase-1</td><td colspan="2">4.1838(2)</td><td>73.233(5)</td><td>/</td><td rowspan="2">3.21</td><td rowspan="2">4.11</td></tr>
<tr><td>phase-2</td><td colspan="2">3.5285(2)</td><td>43.933(3)</td><td>/</td></tr>
</table>

Table S2. Cell parameters for the **cycled cathodes** after thermal treatment **under argon**.

The phase from 25 to 500 °C is hexagonal *α*-$NaFeO_2$ layer structure (*R-3m* space group); *phase-1* is transition metal oxides of cubic structure (*Fm-3m* space group); *phase-2* is nickel or cobalt metal of cubic structure (*Fm-3m* space group). Residuals R_p and R_{wp} do not eliminate the contribution of background fitting but exclude the influence of background noise.

Temperature (°C)		*a* (*b*) (Å)	*c* (Å)	Volume (Å^3)	*c/a*	R_p, %	R_{wp}, %
25		2.8706(0)	14.2238(5)	101.504(4)	4.955	3.51	4.40
250		2.8717(0)	14.2302(5)	101.626(4)	4.955	3.42	4.43
300		2.8736(1)	14.2459(10)	101.876(9)	4.958	3.68	4.84
350		2.8727(1)	14.2253(8)	101.666(8)	4.952	3.27	4.34
400		2.8767(1)	14.2723(9)	102.285(11)	4.961	3.17	4.23
450		2.8734(1)	14.2362(13)	101.792(12)	4.955	3.29	4.51
500		2.8878 (1)	14.2530(15)	102.937(14)	4.936	3.09	3.99
550	*phase-1*	4.1803(7)		73.052(23)	/	3.01	3.83
	phase-2	3.5270(14)		43.875(29)	/		
600	*phase-1*	4.1835(7)		73.221(23)	/	4.26	5.47
	phase-2	3.5250(7)		43.802(12)	/		

Table S3. Cell parameters for the **fresh cathodes** after thermal treatment **under nitrogen**. The phase from 25 to 550 °C is hexagonal α-$NaFeO_2$ layer structure (*R-3m* space group); *phase-1* is transition metal oxides of cubic structure (*Fm-3m* space group); *phase-2* is nickel or cobalt metal of cubic structure (*Fm-3m* space group). Residuals R_p and R_{wp} do not eliminate the contribution of background fitting but exclude the influence of background noise.

<table>
<tr><th colspan="2">Temperature (°C)</th><th>a (b) (Å)</th><th>c (Å)</th><th>Volume (Å^3)</th><th>c/a</th><th>R_p, %</th><th>R_{wp}, %</th></tr>
<tr><td colspan="2">25</td><td>2.8683(0)</td><td>14.2205(4)</td><td>101.301(3)</td><td>4.958</td><td>2.91</td><td>3.76</td></tr>
<tr><td colspan="2">250</td><td>2.8686(0)</td><td>14.2145(3)</td><td>101.297(2)</td><td>4.955</td><td>2.90</td><td>3.66</td></tr>
<tr><td colspan="2">300</td><td>2.8678(0)</td><td>14.2111(3)</td><td>101.220(1)</td><td>4.955</td><td>2.93</td><td>3.69</td></tr>
<tr><td colspan="2">350</td><td>2.8687(0)</td><td>14.2135(3)</td><td>101.296(2)</td><td>4.955</td><td>2.86</td><td>3.68</td></tr>
<tr><td colspan="2">400</td><td>2.8695(0)</td><td>14.2149(3)</td><td>101.366(2)</td><td>4.953</td><td>2.96</td><td>3.84</td></tr>
<tr><td colspan="2">450</td><td>2.8703(0)</td><td>14.2198(4)</td><td>101.456(4)</td><td>4.954</td><td>3.40</td><td>4.41</td></tr>
<tr><td colspan="2">500</td><td>2.8687(0)</td><td>14.2096(4)</td><td>101.271(2)</td><td>4.953</td><td>2.84</td><td>3.69</td></tr>
<tr><td colspan="2">550</td><td>2.8812(1)</td><td>14.24629(23)</td><td>102.416(19)</td><td>4.945</td><td>3.45</td><td>4.87</td></tr>
<tr><td rowspan="2">600</td><td>phase-1</td><td colspan="2">4.1791(2)</td><td>72.987(6)</td><td>/</td><td rowspan="2">2.94</td><td rowspan="2">3.79</td></tr>
<tr><td>phase-2</td><td colspan="2">3.5285(2)</td><td>43.933(4)</td><td>/</td></tr>
</table>

Table S4. Cell parameters for the **cycled cathodes** after thermal treatment **under nitrogen**. The phase from 25 to 500 °C is hexagonal α-$NaFeO_2$ layer structure (*R-3m* space group); *phase-1* is transition metal oxides of cubic structure (*Fm-3m* space group); *phase-2* is nickel or cobalt metal of cubic structure (*Fm-3m* space group). Residuals R_p and R_{wp} do not eliminate the contribution of background fitting but exclude the influence of background noise.

<table>
<tr><th colspan="2">Temperature (°C)</th><th>a (b) (Å)</th><th>c (Å)</th><th>Volume (Å^3)</th><th>c/a</th><th>R_p, %</th><th>R_{wp}, %</th></tr>
<tr><td colspan="2">25</td><td>2.8706(0)</td><td>14.2238(5)</td><td>101.504(4)</td><td>4.955</td><td>3.51</td><td>4.40</td></tr>
<tr><td colspan="2">250</td><td>2.8709(0)</td><td>14.2248(3)</td><td>101.534(3)</td><td>4.955</td><td>3.07</td><td>3.88</td></tr>
<tr><td colspan="2">300</td><td>2.8715(0)</td><td>14.2261(4)</td><td>101.585(3)</td><td>4.954</td><td>3.07</td><td>3.98</td></tr>
<tr><td colspan="2">350</td><td>2.8729(1)</td><td>14.2367(5)</td><td>101.761(5)</td><td>4.955</td><td>3.19</td><td>4.09</td></tr>
<tr><td colspan="2">400</td><td>2.8754(1)</td><td>14.2629(3)</td><td>102.123(7)</td><td>4.960</td><td>3.61</td><td>4.91</td></tr>
<tr><td colspan="2">450</td><td>2.8754(2)</td><td>14.2579(20)</td><td>102.093(19)</td><td>4.959</td><td>3.59</td><td>4.80</td></tr>
<tr><td colspan="2">500</td><td>2.8755(1)</td><td>14.2283(16)</td><td>101.881(14)</td><td>4.948</td><td>3.64</td><td>5.28</td></tr>
<tr><td rowspan="2">550</td><td>phase-1</td><td colspan="2">4.1820(3)</td><td>73.139(8)</td><td>/</td><td rowspan="2">3.37</td><td rowspan="2">4.30</td></tr>
<tr><td>phase-2</td><td colspan="2">3.5265(7)</td><td>43.858(6)</td><td>/</td></tr>
<tr><td rowspan="2">600</td><td>phase-1</td><td colspan="2">4.18063(2)</td><td>73.068(6)</td><td>/</td><td rowspan="2">3.08</td><td rowspan="2">4.91</td></tr>
<tr><td>phase-2</td><td colspan="2">3.52389(2)</td><td>43.759(4)</td><td>/</td></tr>
</table>

Table S5. Cell parameters for the **fresh cathodes** after thermal treatment **under hydrogen**. The phase from 25 to 350 ℃ is hexagonal *α*-$NaFeO_2$ layer structure (*R-3m* space group). Residuals R_p and R_{wp} do not eliminate the contribution of background fitting but exclude the influence of background noise.

Temperature (℃)	*a* (*b*) (Å)	*c* (Å)	Volume (Å^3)	*c/a*	R_p, %	R_{wp}, %
25	2.8683(0)	14.2205(4)	101.301(3)	4.958	2.91	3.76
250	2.8690(0)	14.2175(4)	101.350(4)	4.956	3.12	3.95
300	2.8765(1)	14.2585(17)	102.169(15)	4.957	3.47	4.94
350	2.9017(4)	14.3963(51)	104.976(42)	4.961	3.84	5.79

Table S6. Cell parameters for the **cycled cathodes** after thermal treatment **under hydrogen**. The phase from 25 to 300 ℃ is hexagonal *α*-$NaFeO_2$ layer structure (*R-3m* space group). Residuals R_p and R_{wp} do not eliminate the contribution of background fitting but exclude the influence of background noise.

Temperature (℃)	*a* (*b*) (Å)	*c* (Å)	Volume (Å^3)	*c/a*	R_p, %	R_{wp}, %
25	2.8706(0)	14.2238(5)	101.504(4)	4.955	3.51	4.40
250	2.8754(1)	14.2572(14)	102.082(34)	4.958	3.69	5.30
300	2.8790(3)	14.2847(34)	102.538(30)	4.962	3.91	5.54

Table S7. Cell parameters for the **fresh cathodes** after thermal treatment **under air**.

The phase from 25 to 600 ℃ is hexagonal α-$NaFeO_2$ layer structure (*R-3m* space group). Residuals R_p and R_{wp} do not eliminate the contribution of background fitting but exclude the influence of background noise.

Temperature (℃)	*a* (*b*) (Å)	*c* (Å)	Volume (Å^3)	*c/a*	R_p, %	R_{wp}, %
25	2.8683(0)	14.2205(4)	101.301(3)	4.958	2.91	3.76
250	2.8684(0)	14.2141(3)	101.250(3)	4.955	2.84	3.56
300	2.8690 (0)	14.2168(3)	101.342(4)	4.955	2.92	3.68
350	2.8694(0)	14.2185(3)	101.382(3)	4.955	2.87	3.60
400	2.8695(0)	14.2169(4)	101.377(3)	4.955	2.98	3.77
450	2.8691(0)	14.2145(3)	101.336(3)	4.954	2.97	3.76
500	2.8711(6)	14.2168(29)	101.489(39)	4.952	3.34	4.64
550	2.8696(0)	14.2151(4)	101.370(4)	4.954	3.23	4.16
600	2.8704(0)	14.2195(4)	101.459(4)	4.954	3.10	3.98

Table S8. Cell parameters for the **cycled cathodes** after thermal treatment **under air**.

The phase from 25 to 600 °C is a hexagonal *α*-$NaFeO_2$ layer structure (*R-3m* space group). Residuals R_p and R_{wp} do not eliminate the contribution of background fitting but exclude the influence of background noise.

Temperature (°C)	*a* (*b*) (Å)	*c* (Å)	Volume (Å^3)	*c/a*	R_p, %	R_{wp}, %
25	2.8706(0)	14.2238(5)	101.504(4)	4.955	3.51	4.40
250	2.8709(0)	14.2299(4)	101.570(3)	4.957	3.54	4.55
300	2.8713(0)	14.2369(6)	101.648(4)	4.958	3.44	4.41
350	2.8726(0)	14.2266(7)	101.669(4)	4.952	3.29	4.42
400	2.8725(1)	14.2224(9)	101.627(8)	4.951	3.72	4.87
450	2.8731(1)	14.2223(10)	101.674(8)	4.950	3.55	4.87
500	2.8746(1)	14.2295(10)	101.833(8)	4.950	3.17	4.15
550	2.8760(1)	14.2310(10)	101.933(8)	4.948	3.36	4.80
600	2.8745(1)	14.2313(12)	101.832(10)	4.951	3.55	4.86

Table S9. Cell parameters for the **fresh cathodes** after thermal treatment **under oxygen**.
The phase from 25 to 600 ℃ is hexagonal α-$NaFeO_2$ layer structure (*R-3m* space group). Residuals R_p and R_{wp} do not eliminate the contribution of background fitting but exclude the influence of background noise.

Temperature (℃)	*a* (*b*) (Å)	*c* (Å)	Volume (Å^3)	*c/a*	R_p, %	R_{wp}, %
25	2.8683(0)	14.2205(4)	101.301(3)	4.958	2.91	3.76
250	2.8701(0)	14.2210(5)	101.421(4)	4.955	3.38	4.48
300	2.8684(0)	14.2144(3)	101.282(2)	4.956	3.03	3.82
350	2.8695(0)	14.2146(3)	101.365(2)	4.954	2.98	3.82
400	2.8691(0)	14.2201(3)	101.455(2)	4.956	2.17	3.14
450	2.8705(0)	14.2263(4)	101.415(4)	4.956	2.96	3.83
500	2.8726(1)	14.2260(10)	101.661(8)	4.953	3.34	4.79
550	2.8724(1)	14.2273(10)	101.657(10)	4.953	3.41	4.77
600	2.8680(4)	14.2159(5)	101.266(4)	4.957	3.15	4.17

Table S10. Cell parameters for the **cycled cathodes** after thermal treatment **under oxygen**. The phase from 25 to 600 ℃ is hexagonal *α*-$NaFeO_2$ layer structure (*R-3m* space group). Residuals R_p and R_{wp} do not eliminate the contribution of background fitting but exclude the influence of background noise.

Temperature (°C)	*a* (*b*) (Å)	*c* (Å)	Volume (Å^3)	*c/a*	R_p, %	R_{wp}, %
25	2.8706(0)	14.2238(5)	101.504(4)	4.955	3.51	4.40
250	2.8701(0)	14.2210(4)	101.449(4)	4.955	3.22	4.13
300	2.8695(0)	14.2185(4)	101.393(4)	4.955	3.14	4.14
350	2.8698(0)	14.2194(5)	101.418(5)	4.955	3.25	4.39
400	2.8711(0)	14.2211(5)	101.676(4)	4.953	2.40	3.40
450	2.8725(1)	14.2267(8)	101.664(6)	4.953	3.50	4.91
500	2.8712(0)	14.2331(4)	101.359(4)	4.956	2.99	3.98
550	2.8693(0)	14.2213(4)	101.346(5)	4.956	3.26	4.31
600	2.8728(1)	14.2272(8)	101.684(7)	4.952	3.35	4.68

Table S11. Elemental compositions from ICP-OES and TECG analyses of the obtained active material after thermal treatment under air at different temperatures.

Avg: Average; SD: Standard deviation.

Temperature (°C)	*Weight percent (%)*											
	Ni		Co		Mn		Li		**Al**		O	
	Avg	SD	Avg	SD	Avg	SD	Avg	SD	**Avg**	**SD**	Avg	SD
400	30.6	0.5	11.6	0.3	10.9	0.2	6.7	0.08	**0.46**	**0.013**	35.3	0.7
450	31.0	1.0	11.8	0.3	11.1	0.2	6.8	0.12	**0.35**	**0.005**	35.6	0.7
500	30.3	0.2	12.0	0.2	11.3	0.1	7.0	0.03	**0.29**	**0.021**	36.3	0.7

Table S12. Elemental compositions from ICP-OES and TECG analyses of the obtained active material after thermal treatment under air at 450 °C with and without water-ethanol washing.

Avg: Average; SD: Standard deviation.

	Weight percent (%)									
	Ni		Co		Mn		**Li**		O	
	Avg	SD	Avg	SD	Avg	SD	**Avg**	**SD**	Avg	SD
Without washing	31.8	0.4	11.4	0.2	10.7	0.1	**6.88**	**0.07**	34.5	0.5
With washing	31.8	0.7	12.2	0.1	11.5	0.1	**6.02**	**0.06**	36.8	0.3

Table S13. Cell parameters for the **fresh anodes** after thermal treatment **under argon**.

The phase from 25 to 600 °C is hexagonal layer structure (*$P6_3/mmc$* space group). Residuals R_p and R_{wp} do not eliminate the contribution of background fitting but exclude the influence of background noise.

Temperature (°C)	*a* (*b*) (Å)	*c* (Å)	Volume (Å^3)	R_p, %	R_{wp}, %
25	2.4615(5)	6.7141(21)	35.229(16)	5.13	8.00
250	2.4606(5)	6.7105(23)	35.187(17)	6.84	9.93
300	2.4592(6)	6.70737(28)	35.130(20)	5.69	8.55
350	2.4626(5)	6.71644(25)	35.275(17)	5.99	7.85
400	2.4602(5)	6.7091(25)	35.168(18)	5.90	8.21
450	2.4593(4)	6.7080(22)	35.136(14)	5.90	8.21
500	2.4590(5)	6.7069(23)	35.120(17)	6.17	8.71
550	2.4603(6)	6.7112(30)	35.179(20)	7.35	11.2
600	2.4599(5)	6.7096(22)	35.161(15)	6.69	9.61

Table S14. Cell parameters for the **cycled anodes** after thermal treatment **under argon**.

The phase from 25 to 600 °C is a hexagonal layer structure (*P*6_3*/mmc* space group). Residuals R_p and R_{wp} do not eliminate the contribution of background fitting but exclude the influence of background noise.

Temperature (°C)	*a* (*b)* (Å)	*c* (Å)	Volume (Å^3)	R_p, %	R_{wp}, %
25	2.4610(4)	6.7130(16)	35.210(11)	4.82	7.47
250	2.4595(12)	6.7108(60)	35.154(38)	4.47	6.15
300	2.4585(5)	6.7069(24)	35.107(16)	4.18	5.77
350	2.4607(10)	6.7136(49)	35.203(33)	5.46	9.74
400	2.4606(4)	6.7121(21)	35.194(12)	5.08	7.80
450	2.4592(18)	6.70810(84)	35.134(6)	4.55	6.39
500	2.4613(5)	6.7135(22)	35.220(15)	5.48	10.7
550	2.4593(6)	6.7092(27)	35.141(18)	4.58	6.50
600	2.4604(10)	6.7133(49)	35.195(35)	5.53	9.69

Table S15. Cell parameters for the **fresh anodes** after thermal treatment **under nitrogen**. The phase from 25 to 600 °C is a hexagonal layer structure (*$P6_3/mmc$* space group). Residuals R_p and R_{wp} do not eliminate the contribution of background fitting but exclude the influence of background noise.

Temperature (°C)	*a* (*b*) (Å)	*c* (Å)	Volume (Å^3)	R_p, %	R_{wp}, %
25	2.4615(5)	6.7141(21)	35.229(16)	5.13	8.00
250	2.4599(3)	6.70812(13)	35.152(10)	4.54	6.52
300	2.4595(4)	6.7095(17)	35.150(13)	5.06	8.12
350	2.4596(2)	6.7099(9)	35.154(7)	4.06	5.63
400	2.4598(4)	6.7100(16)	35.159(17)	4.71	7.12
450	2.4607(5)	6.7108(27)	35.190(18)	5.27	8.53
500	2.4605(5)	6.7111(27)	35.185(19)	5.57	8.78
550	2.4614(7)	6.7140(4)	35.226(24)	4.46	6.53
600	2.4603(3)	6.7106(12)	35.176(7)	4.60	6.84

Table S16. Cell parameters for the **cycled anodes** after thermal treatment **under nitrogen**. The phase from 25 to 600 ℃ is a hexagonal layer structure (*$P6_3/mmc$* space group). Residuals R_p and R_{wp} do not eliminate the contribution of background fitting but exclude the influence of background noise.

Temperature (℃)	*a* (*b*) (Å)	*c* (Å)	Volume (Å^3)	R_p, %	R_{wp}, %
25	2.4610(4)	6.7130(16)	35.210(11)	4.82	7.47
250	2.4598(5)	6.7108(23)	35.163(14)	4.94	7.31
300	2.4610(4)	6.7125(22)	35.206(15)	4.30	5.88
350	2.4595(4)	6.7104(20)	35.154(14)	4.46	6.57
400	2.4601(6)	6.7095(30)	35.167(20)	4.30	5.83
450	2.4618(6)	6.7154(27)	35.246(11)	4.46	6.60
500	2.4609(7)	6.7136(36)	35.209(25)	6.02	11.00
550	2.4596(6)	6.7109(28)	35.160(18)	5.07	7.96
600	2.4611(7)	6.7148(33)	35.223(21)	5.67	9.44

Table S17. Cell parameters for the **fresh anodes** after thermal treatment **under hydrogen**. The phase from 25 to 600 °C is a hexagonal layer structure (*P6₃/mmc* space group). Residuals R_p and R_{wp} do not eliminate the contribution of background fitting but exclude the influence of background noise.

Temperature (°C)	*a* (*b)* (Å)	*c* (Å)	Volume (Å^3)	R_p, %	R_{wp}, %
25	2.4615(5)	6.7141(21)	35.229(16)	5.13	8.00
250	2.4593(7)	6.7083(28)	35.137(20)	5.74	9.30
300	2.4591(8)	6.7088(36)	35.134(24)	6.08	10.4
350	2.4611(6)	6.71382(28)	35.217(17)	6.04	9.96
400	2.4594(6)	6.7094(28)	35.146(19)	5.92	9.73
450	2.4639(7)	6.7079(35)	35.265(22)	6.13	9.30
500	2.4608(4)	6.7104(11)	35.190(12)	6.34	9.66
550	2.4590(8)	6.7092(40)	35.133(27)	4.89	7.33
600	2.4590(5)	6.7072(20)	35.121(16)	6.16	10.50

Table S18. Cell parameters for the **cycled anodes** after thermal treatment **under hydrogen**. The phase from 25 to 600 °C is a hexagonal layer structure (*P6₃/mmc* space group). Residuals R_p and R_{wp} do not eliminate the contribution of background fitting but exclude the influence of background noise.

Temperature (°C)	*a* (*b)* (Å)	*c* (Å)	Volume (Å^3)	R_p, %	R_{wp}, %
25	2.4610(4)	6.7130(16)	35.210(11)	4.82	7.47
250	2.4591(10)	6.70901(46)	35.156(32)	6.00	9.96
300	2.4596(12)	6.7105(56)	35.156(36)	5.80	9.63
350	2.4601(15)	6.71175(73)	35.177(50)	6.84	11.50
400	2.4628(17)	6.7260(84)	35.331(55)	7.69	15.00
450	2.4594(7)	6.7103(32)	35.150(24)	8.12	12.60
500	2.4597(12)	6.7109(54)	35.163(36)	8.27	13.00
550	2.4598(4)	6.7111(16)	35.167(12)	5.45	7.75
600	2.4592(7)	6.7110(33)	35.149(21)	5.66	9.17
